# Bimodality in *E. coli* gene expression: Sources and robustness to genome-wide stresses

**DOI:** 10.1371/journal.pcbi.1012817

**Published:** 2025-02-13

**Authors:** Ines S. C. Baptista, Suchintak Dash, Amir M. Arsh, Vinodh Kandavalli, Carlo Maria Scandolo, Barry C. Sanders, Andre S. Ribeiro

**Affiliations:** 1 Faculty of Medicine and Health Technology, Tampere University, Tampere, Finland; 2 Department of Cell and Molecular Biology, Uppsala University, Uppsala, Sweden; 3 Department of Mathematics & Statistics, University of Calgary, Calgary, Canada; 4 Institute for Quantum Science and Technology, University of Calgary, Calgary, Canada; Mount Sinai School of Medicine: Icahn School of Medicine at Mount Sinai, INDIA

## Abstract

Bacteria evolved genes whose single-cell distributions of expression levels are broad, or even bimodal. Evidence suggests that they might enhance phenotypic diversity for coping with fluctuating environments. We identified seven genes in *E. coli* with bimodal (low and high) single-cell expression levels under standard growth conditions and studied how their dynamics are modified by environmental and antibiotic stresses known to target gene expression. We found that all genes lose bimodality under some, but not under all, stresses. Also, bimodality can reemerge upon cells returning to standard conditions, which suggests that the genes can switch often between high and low expression rates. As such, these genes could become valuable components of future multi-stable synthetic circuits. Next, we proposed models of bimodal transcription dynamics with realistic parameter values, able to mimic the outcome of the perturbations studied. We explored several models’ tunability and boundaries of parameter values, beyond which it shifts to unimodal dynamics. From the model results, we predict that bimodality is robust, and yet tunable, not only by RNA and protein degradation rates, but also by the fraction of time that promoters remain unavailable for new transcription events. Finally, we show evidence that, although the empirical expression levels are influenced by many factors, the bimodality emerges during transcription initiation, at the promoter regions and, thus, may be evolvable and adaptable.

## Author summary

Bacterial cell populations exhibiting multiple phenotypes under the same environmental condition should have enhanced survival chances to unpredictable environmental fluctuations. This capacity could rely on genes with the ability to switch between high- and low-expression levels, under the same environment. So far, few genes are known to have such a capacity. We report on seven genes with bimodal dynamics, show that bimodality is robust to some but not all stresses, and reversibility of bimodality if returning to control conditions. We also show evidence that bimodality emerges from events in transcription initiation and propose a model of their behavior. Our findings could advance research on bacterial decision-making processes and engineering multi-stable synthetic circuits.

## 1. Introduction

Environmental changes modify which, when, and how cell resources should be used to maximize survivability and growth. For example, bacteria, when in richer media, usually opt for accelerating growth (which requires the quick consumption of resources). Meanwhile, in poor media, they favor using internal resources for survival alone by reducing growth rates. Quick adaptability to environmental changes by tuning resources’ usage is essential to outcompete other bacteria and survive stresses [1–4].

Bacteria have evolved transcriptional programs that implement phenotypically advantageous modifications following environmental changes [5–10]. To support this, they also evolved stress-sensing mechanisms that, when activated, trigger these transcriptional programs. Nevertheless, in some cases, stochastic switching between phenotypes can be favored over sensing [11]. In other cases, the changes might be relatively quick and stochastic in nature, and/or cells lack sufficient time and/or information to make informed decisions.

Evidence suggests that, in these scenarios, isogenic cell populations can increase their survivability by increasing phenotypic heterogeneity, based on an inheritable ability to express multiple distinct phenotypes [4,11–19]. This can be made possible by genes that can be expressed at two (or more) distinct levels, under the same conditions.

Knowledge on bimodal gene expression systems in isogenic bacterial populations has been growing for several years. [20] reported stable bimodal single-cell expression levels for P_araBAD_, potentially due to active uptake of the inducer. [21] reported a nutrient-tunable bistable switch in *S. enterica* (that differs over time and with the nutrient concentration). [22] reported that the expression of *lasB* in *P. aeruginosa* is bimodal and responsive to population density. [23] reported a stimuli-responsive system of five bimodal operons (*LEE1* to *LEE5*) in an enteropathogenic *E. coli*. Moreover, [24] reported the existence of a few bimodal distributions (non-specified) albeit rare and not visually clear. Meanwhile, [25] reported a feedback loop controlled-bimodal expression in *B. subtilis*. Finally, [26] showed that well-mixed planktonic cultures of *E. coli* exhibit bimodal activation of curli gene expression, causing stochastic differentiation into subpopulations of curli-positive and curli-negative cells. Similar systems have been reported in higher order organisms as well, including yeast [27,28].

Bimodality has also been explored using theoretical models. These models predict that bimodality can emerge from the internal properties of the gene network, such as transcription factor (TF) binding, both cooperative [29] as well as non-cooperative [30]. It can also emerge from feedback loops, as well as by tuning other internal properties [31–33]. For a review of internal mechanisms that can generate gene expression bimodality [34,35]. Other works explored the influence of the environment [12], including a recent model inspired by quantum resource theories [36].

The phenotypic advantages of genes with bimodal expression should depend on their sensitivity to specific signals, as well as their robustness to other, non-related perturbations, as it will influence their potential usability in future synthetic genetic circuits. Here, we investigated the robustness of seven genes of the genome of *E. coli*, which we found to have bimodal single-cell expression levels during standard growth conditions. For this, we used strains carrying the proteins fused with YFP, which we obtained from the library [24]. We subjected each strain to several stresses and measured changes in their bimodal, single-cell gene expression distributions. From the data, we studied the propensity to shift from being bimodal to unimodal under the stress conditions, asymmetries in the single cell distributions, and the recoverability of bimodality. We also discussed the sources of bimodality. In the end, we propose an empirically based stochastic models of bimodal gene expression, which account for the effects of perturbations, which we used to further explore the robustness of bimodalities assuming different means by which the bimodality can shift to unimodality.

## 2. Results

### 2.1. Empirical distributions of single-cell protein levels

A previous work [37] investigated single-cell expression levels of genes during the exponential and the stationary growth phases as a function of promoter σ factor preference. That study proposed a predictive model of changes in expression level during growth-phase transition as a function of the promoter sequence. Whereas mean and variability of single-cell expression levels was investigated, the modes of the single-cell distributions of expression levels were not studied. Subsequent data analysis revealed that two genes (*metK* and *pgi*) exhibited distributions of single-cell expression levels that could potentially be bimodal.

**Fig 1:**
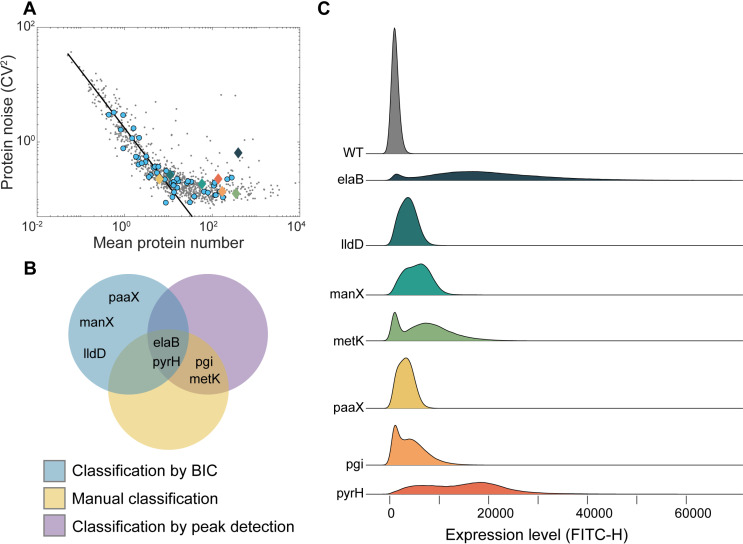
(A) Scatter plot between mean and noise (squared coefficient of variation, CV^2^) in single-cell protein numbers of the data reported in [24]). Blue balls are the 49 genes with unimodal dynamics. The remaining seven genes were found to have ‘bimodal dynamics’ and are highlighted and colored in accordance with the colors of the single-cell distributions in (C). The black line is the best fitting y=Cx equation. **(B)** Classification of genes as having bimodal expression using manual classification, Bayesian information criterion (BIC), and peak detection, respectively. Shown is a Venn Diagram depicting which genes were classified as bimodal and by which criteria. **(C)** Best fits of the empirical single-cell distributions of protein levels of each of the seven genes under standard growth conditions that were classified as bimodal by at least one criterion. Each distribution results from merging three biological repeats (Fig D in S1 Text). Also shown is the distribution of the wild type (WT) strain (grey), which does not code for any YFP. The color scheme facilitates data visualization.

To find additional genes with significant chances of exhibiting bimodal dynamics, we considered the known mean and noise of single-cell protein numbers reported for 1018 genes of *E. coli* during standard growth conditions [24] (Fig 1A). Then, we selected 56 genes along a large portion of the space state of the mean over the noise in single-cell protein levels. Of these 56 genes, 70% were selected for having relatively high noise (i.e., are above the best fitting line in Fig 1A) reported in [24]. The remaining genes were selected for the opposite reason, for comparison. Relevantly, 6 out of the 7 genes found to have bimodal distributions are located above the line. Fig A in S1 Text shows how our measurements of these genes’ expression levels compare to the levels reported in [24].

Next, using flow cytometry, we obtained single-cell distributions of protein levels for each of the 56 genes, in standard growth conditions (Section 1.1 and 1.2 in S1 Text). We found that 7 of the 56 genes exhibited bimodal distributions, according to at least one of the criteria in Section 1.4 in S1 Text (Fig 1B).

Fig 1C shows the best fits to the merged single-cell distributions of protein levels for each of the seven genes and corresponding strains (Section 1.5, histograms plotted in Fig B in S1 Text). Specifically, for each gene, the distribution results from merging the data from three biological replicates. Also shown is the data corresponding to the wild-type (WT) strain. Data for each replicate is provided in S1 Text. From here onwards, for simplicity, we refer to the fits as “distributions” as well.

Visibly, the distributions differ significantly between genes. For example, arguably, whereas for *elaB*, *metK* and *pyrH*, the peaks of the two modes are very distant from each other, large distance is not evident for *manX*, *paaX*, and *lldD*. Also, whereas in *elaB*, *metK* and *pgi*, the “choice of mode” by each cell is highly asymmetric, for the other genes the distribution looks symmetric (same chances for both modes). Meanwhile, the distributions from each replicate are shown in Figs C and D in S1 Text. Visibly, there is little difference between replicates, suggesting that the bimodality is stable under stable conditions.

Notably, all distributions are asymmetric (i.e., right sided), with the ‘proportion’ of the total areas related to the right mode being larger than the proportion related to the left mode. Whereas ‘higher peaks’ in the left modes could potentially be explained by the boundary at zero, this asymmetry likely cannot.

One could hypothesize that the low-fluorescence intensity values of the first mode of the distributions in Fig 1C result from unhealthy or dead cells, rather than from healthy cells with low expression levels. To test this hypothesis, we compared the normalized single-cell distribution of fluorescent levels of each strain (FITC-H, commonly used as proxies for protein numbers) with the corresponding normalized single-cell distributions of pulse width, SSC-H, and FSC-H, respectively (parameters commonly used as proxies of cell size) [38]. From Figs E–G in S1 Text, we found little to no similarity between the distributions. Visibly, the peaks are not aligned, and the mode weights are not similar. Only rarely is there similarity, but never in all biological replicates of a given strain. These results are line with [24], which reported that these strains are as healthy as the other strains of the YFP library. This is supported by the density scatter plots between the single-cell protein fluorescence levels and the pulse width, SSC-H, and FSC-H, respectively (Figs H–J in S1 Text). In these, while some correlations are observed between YFP intensity and the cell morphology parameters (which is expected, since cells often regulate their protein concentrations rather than total numbers), no concurrent bimodality is evident in these channels.

Also noteworthy, the shapes of the first modes of the distributions differ widely between strains (Fig 1C), e.g. in mean values and in fraction of cells in that mode. We use this as evidence that the fractions of the cells with low or absent fluorescence levels differ and the levels themselves also differ, suggesting that they do not result from the same cause. Moreover, from microscopy images, we did not find differences in cell morphology (i.e., length, width and area) that could explain the bimodal gene expression such as asymmetric in cell division (Fig K in S1 Text). Also, we found no biased spatial distributions of YFP tagged proteins (Fig L in S1 Text).

Given the above, we conclude that the bimodal distributions of single-cell protein fluorescence levels cannot be explained by health or morphology features of these strains, which would cause a significant fraction of cells to behave similarly to wild-type cells. Instead, the bimodal distributions likely result from each cell population being able to express the tagged proteins at two distinct rates (which differ between strains).

In this regard, from Fig 1C, in some strains, particularly *pyrH*, *paaX* and *manX*, cells composing the first mode of distribution appear to have low expression levels (Table A in S1 Text). In the other strains, the cells exhibit fluorescence levels similar to the WT strain, suggesting that the genes are not active.

### 2.2. Stresses responsiveness and robustness

Next, we investigated the robustness of the bimodality of each of the seven genes to five different stresses. Noteworthy, these stresses have direct, as well as indirect effects on gene expression. For example, cold shock is expected to have multiple effects, from influencing DNA supercoiling to cellular energy, etc. [10].

First, we studied the effects of the transitioning from exponential to stationary growth. During this transition, cells actively increase the number of RpoS (σ^38^), activating ~10% of the genome [39–42]. Moreover, since RNA polymerase (RNAP) numbers are lower than σ factor numbers, many genes not recognized by σ^38^ become less expressed [43–45]. Meanwhile, *lldD*, *manX*, *paaX*, and *pyrH* are σ^70^-dependent [46] and, thus, are expected to be less expressed during this growth phase.

**Fig 2:**
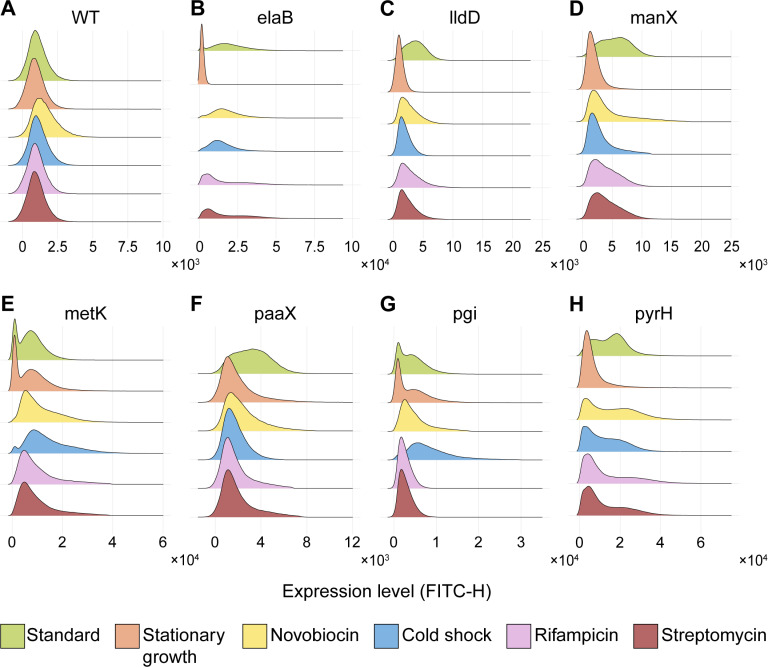
Robustness of gene expression bimodality to perturbations targeting gene expression. Best fits of the single-cell distributions of protein expression of genes classified as bimodal in standard growth conditions (green), when subjected to stationary growth (orange), novobiocin (yellow), cold shock (blue), rifampicin (pink) and streptomycin (brown), respectively. The control (WT, absence of genes expressing YFP) is shown for comparison. Each distribution results from merging the data from three biological repeats.

Results are shown in Fig 2 (orange distributions). Compared to the control condition (green distributions), we find that most genes (*lldD*, *manX*, *paaX*, *pyrH*) were strongly repressed, as expected. Namely, their distributions of expression levels appear to become unimodal, in the region originally occupied by the lower peak of the bimodal distribution. This would imply that the lower expression “state”, already existent during standard growth, now became dominant.

Another possible explanation is that the system remains bistable, but its two states do not differ sufficiently in expression levels to be distinguishable. This would occur if, e.g., the differences between the mean protein numbers of the two states are smaller than the variabilities.

Meanwhile, *metK* and *pgi* are not repressed, maintaining bimodality, which is consistent with them being dual σ^70^ and σ^38^-dependent [37]. Finally, *elaB* was also repressed during the growth phase transition, albeit being reported to be σ^38^-dependent [46]. Relevantly, high expression of this gene during exponential growth was also reported in [24]. This may explainable by its negative regulation by *rob*, whose expression might be enhanced during the stationary growth phase [47]). Another explanation would be the dual regulation by *oxyR* [46].

Second, we perturbed the processes regulating DNA supercoiling. We used Novobiocin, an antibiotic that binds to the GyrB subunit of DNA gyrase, which is needed to resolve positive supercoiling buildup (PSB), blocking ATPase activity [48,49]. We expect the responsiveness of the genes, and, thus, changes in their bimodality, to differ with their supercoiling sensitivity. A past study reported that *lldD*, *manX*, *metK*, and *pgi* are highly sensitive to positive supercoiling buildup during standard growing conditions [10]. Meanwhile, from Fig 2, these are the genes whose activity was, comparatively, more reduced, with *pyrH* being the only one maintaining bimodality.

Third, we subjected cells to cold shock, which hampers gene expression in multiple ways, including the disruption of PSB [10,50,51]. In addition, cold shock is expected to influence transcription directly, particularly the initiation process [50,52]. Other effects include hampering diffusion in the cytoplasm [53] and indirectly causing significant decreases in cellular ATP levels [10]. As such, effects on individual genes are not easily predictable. To evaluate the consistency of the results we compared them with the results under Novobiocin. Past studies showed that nearly half of cold shock repressed genes are also highly responsive to gyrase inhibition [10]. In agreement, we find that the genes show similar responses, except *pgi* and *metK*. For example, the genes remaining bimodal under cold shock and under novobiocin are *elaB* and *pyrH*. Meanwhile, only *metK* and *pgi* remains bimodal under cold shock alone, and only *manX* remains bimodal under novobiocin alone.

Fourth, we hampered transcription rates. For this, we used rifampicin, an antibiotic that binds to the β sub-unit of RNAP [54] and prevents RNAP from escaping beyond 2–3 nucleotides away from the TSS [55,56]. In agreement, we find that all genes have weaker expressions than during standard growth.

Finally, we targeted translation by adding Streptomycin, an antibiotic that binds to the ribosome’s 30S subunit [57] and affects the ability of ribosomes to decode the RNA [58], which results in the formation of non-functional proteins [59]. Given the strong correlation between RNA and protein numbers in *E. coli* [60], one would expect that this antibiotic should cause similar effects to rifampicin, which agrees with what we observe (Fig 2).

Overall, qualitatively, we observed a wide variety of responses to the stresses. Different genes lose their bimodality to different perturbations, with 4 of 7 of the genes losing their bimodality to all perturbations. Interestingly, no perturbation forced all genes to lose bimodality. These results suggest that the genes of *E. coli* with bimodal gene expression studied here exhibit responsiveness to some stresses but also robustness to other stresses targeting gene expression.

### 2.3. Changes in the shapes of the bimodal distributions with the stresses

To quantify how the perturbations targeting gene expression affected the shape of the bimodal distributions, we considered the following shape parameters:

(i) Distance between peaks 1 and 2 (|*pk*_*1*_ *- pk*_*2*_|) as a measure of the difference between the two possible ‘states’ of gene expression (high and low expression).
(ii) Difference between peak heights (i.e., between the PDF values at the peaks’ positions, *PDF*_*2*_ - *PDF*_*1*_) as a measure of the asymmetry in the proportions of subpopulations in different states.
(iii) Fraction of cells whose fluorescence level is in between the two peaks (*n*_*overlap*_), is used as an indirect measure (positively correlated) of the frequency with which cells may be switching between states and/or for the stochasticity of either (or both) states. However, it needs to be considered that, instead, they may result from broad distributions with complex shapes.

To compare between genes, we next defined relative shape parameters (illustrated in Fig M in S1 Text). We calculated the relative distance between peaks (*d*) by normalizing the above difference with the entire range of the distribution (Eq. 1). Likewise, we calculated the relative difference between peaks height (*h*) by normalizing the difference by the maximum height of the distribution (Eq. 2). Finally, we obtained a relative overlapping region (*o*) from the number of observations between the two peaks divided by the total amount of observations (*n*_*total*_) (Eq. 3):


$$d=\frac{\left|pk_{2}-pk_{1}\right|}{Distribution\;range}$$ (1)

$$h=\frac{PDF_{1}-PDF_{2}}{{\text{Maximum}}_{PDF}}$$ (2)

$$o=\frac{n_{overlap}}{n_{total}}$$ (3)

Noteworthy, these parameters should only be considered if the distributions are bimodal. Fig N in S1 Text shows *d*, *h*, and *o*, for each distribution in Fig 2 (when a distribution for a given perturbation was not classified as bimodal, it was discarded). Visibly, the perturbations alter each of the features characterizing the shapes and do it differently between genes and between perturbations. These results suggest that it should be possible to externally fine-tune the bimodal distributions since *d*, *h*, and *o* exhibit many different values within a significant range of values rather than, e.g., being binary-like, which would hamper fine-tuning.

### 2.4. Bimodality lost due to cells entering stationary growth phase can reemerge if cells reenter the exponential growth phase

We explored whether the bimodality, after becoming undetectable, can again become detectable. We tested this in the case of the transition to stationary growth phase, since this perturbation (along with streptomycin) was the one causing most genes to lose bimodality. For this, we tracked the same cell culture for three generations, while placing the cells in new media once they reach the late stationary growth phase at each generation. Specifically, at each generation, we measure gene expression by flow-cytometry at four growth phases: early and late exponential, followed by early and late stationary growth (Section 1.2 in S1 Text). With respect to time, the consecutive phases are separated by ~50 min, ~150 min, ~200 min, respectively.

**Fig 3:**
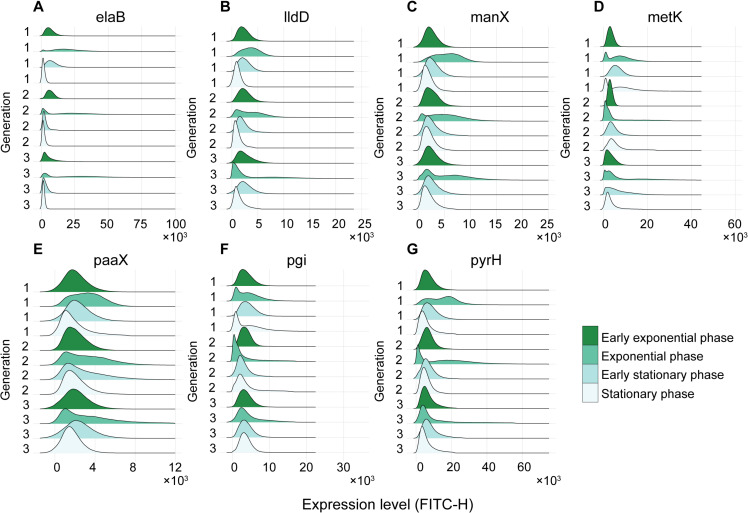
Single-gene expression levels across generations and growth phases. Shown are best fits to the single-cell distributions of expression levels of **(A)** *elaB*, **(B)** *lldD*, **(C)** *manX*, **(D)** *metK*, **(E)** *paaX*, **(F)** *pgi*, and **(G)** *pyrH*. For these measurements, overnight cells are first placed in fresh media, which over time becomes poor, forcing cells to enter stationary growth phase. At that moment, the same cells were inoculated into fresh media, causing them to re-enter the exponential growth phase. The same steps are repeated a second time. Consequently, the same cells go through exponential growth phase three times and stationary growth phase three times as well (Section 1.2 in S1 Text). Measurements are conducted four times, during each generation (early exponential, exponential, early stationary, and stationary phases) resulting in 12 time points.

In Fig 3A–3G, for each strain, we plotted the single-cell distributions of expression levels at each of the four growth phases (early exponential, exponential, early stationary, and stationary), for the three consecutive generations. First, for all genes and in all generations, the shapes of the distributions differ significantly between growth phases. Second, for most genes and phases, the behaviors observed in the first generation (unimodality and bimodality) were also observed in the next generations. For example, for *manX*, the bimodality observed in the late exponential phase (and only in that phase) during the first generation, is again observed in that phase (and only in that phase) in the subsequent generations.

Nevertheless, the shapes of the distributions do, in some cases, differ significantly. There are many possible reasons for this. One explanation may be in the methodology used for the measurements, in that the original media from which we removed cells into fresh media at each generation, was not renewed overtime. Finally, arguably, since the changes in the shape of the distributions occurred over short periods of time (implying that many cells changed state), these modifications can be used as evidence that the genes can “switch state” (between high- and low-expression rates) relatively frequently.

### 2.5. Evidence for hysteresis

Since bimodality in gene expression reemerged when cells were again placed in fresh media (albeit with differences in the shapes of the distributions), we searched for potential hysteresis. Its existence could be evidence for the influence of the systems’ history in its response. This can occur in gene expression patterns across generations when, e.g., there were transcriptome changes during the first adaptation that still remain during the second adaptation [61] and has been previously linked to multistable networks [62].

We tracked the bimodal distributions shape parameters *d* and *o*, to search for hysteresis, of genes with bimodal behaviors in more than one growth condition (*elaB*, *metK*, *pgi*, *pyrH*). We did not track the parameter *h*, as it depends on the fraction of cells extracted between generations and, thus, on the experimental procedure. Nevertheless, as we inoculate the same volume from one generation to the next, this fraction should be relatively similar.

From Fig O in S1 Text, the phase transitions have distinct paths for *d* and *o*, implying that the distribution depends on its history, in agreement with the existence of hysteresis. Nevertheless, in most genes, there is no ‘loop closing’. In these cases, there is no evidence that the cell population returned to its original state, implying the potential existence of only 'partial hysteresis'. Moreover, there are other potential explanations for the observations that will need to be considered in future works.

### 2.6. Bimodality in single cell promoter activity levels

To investigate which mechanisms might be causing bimodality in the expression levels, we next made use of a library of transcriptional fusions of GFP of promoters of *E. coli* [63]. Each promoter fusion is on a low-copy plasmid, and it controls the expression of GFP alone (Section 1.2 in S1 Text). The library includes plasmids with the promoters of 6 out of the 7 genes studied, namely, *elaB*, *manX*, *metK*, *paaX*, *pgi*, and *pyrH*, respectively.

**Fig 4:**
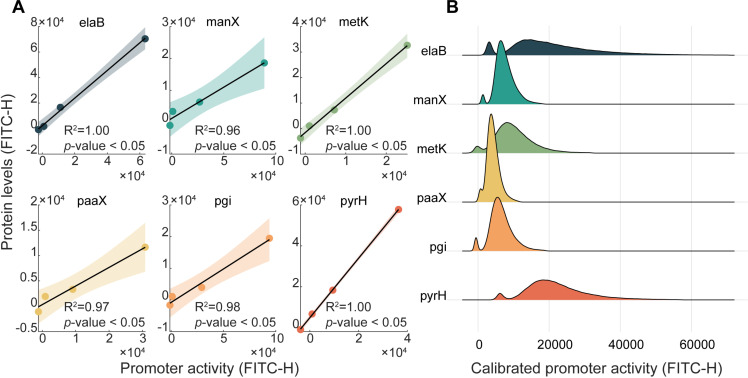
(A) Scatter plot between the levels of YFP expressed by the genes classified as bimodal and GFP expressed by the promoters of those genes on low-copy plasmids. Also shown are the best linear fits along with the corresponding 95% confidence bounds, coefficients of determination (R^2^) and the *p*-values of *t*-tests (Section 1.5 in S1 Text). These lines serve for mapping fluorescence intensities of GFP to fluorescence intensities of YFPs. **(B)** Best fits to the calibrated single-cell distributions of GFP levels expressed from low-copy plasmids [63], under standard growth conditions. Each distribution results from merging the data from three biological repeats. The color scheme facilitates data visualization and the correspondences to the figures in (A), used for the calibration.

We measured the single-cell fluorescent levels of each of the six strains under standard growth conditions. Then, we compared them with the strains of the YFP library (in Fig 1C). For this, we first considered that GFP and YFP differ in fluorescence intensity. Thus, we ‘mapped’ the data from the promoter fusion library into the data from the YFP strain library. For this, for each of the 6 promoters measured, we calibrated 4 data points of the single-cell distributions of fluorescence levels using the strain of the GFP library to the corresponding data points using the strain from the YFP library (Fig 4A). The points were the fluorescence levels of the cell with lowest signal, the cell with highest signal, the cells at the mean of the weak mode, and the cells at the mean of the high mode. Finally, using these points, we estimated the calibrated distribution by linear interpolation. We plotted the calibrated distributions in Fig 4B. Using the peak detection method (Section 1.4 in S1 Text), we find that all calibrated distributions from the GFP library are bimodal, confirming the conclusions using the strains of the YFP library.

Next, we considered that: (i) The promoters controlling GFP expression are in low-copy plasmids, not in the chromosome. As such, being at the chromosome is not essential for the generation of bimodality in single-cell protein levels. Given this, we argue that supercoiling buildup is not an a essential source of bimodality of the genes studied, since the plasmids used lack topological constrains that could allow for strong positive supercoiling buildup [64] (ii) The plasmids only express GFP, not the native protein, and many plasmids of the GFP library do not exhibit bimodal distributions. Thus, events during transcription elongation or termination of the native proteins in the YFP library are not necessary to generate the bimodality observed (albeit these events could influence the shapes of the single-cell distributions of fluorescence levels). (iii) Finally, for the same reasons, we argue that the processes of RNA and protein degradation of the native proteins studied using the YFP library are not necessary to generate the bimodality, as the bimodality still emerges in the corresponding cells of the GFP library.

Overall, since the only structure common to both libraries are the promoter regions, where transcription initiation occurs, we conclude that the bimodality in the single-cell distributions of protein expression levels in cells of both libraries is generated by events that occur at the promoter region.

### 2.7. Reduced stochastic model of bimodal gene expression

We developed stochastic models of bimodal gene expression to mimic the responsiveness to the perturbations studied above. We use the models to explore the state space, in order to identify influential parameter values and find saturation points.

**Fig 5:**
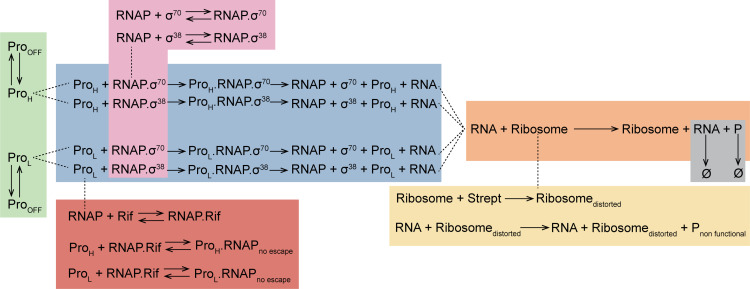
Detailed model of gene expression that includes transcription locking due to PSB (green region), σ factor regulation (pink region), and translation (orange region). Also included are effects of rifampicin, which hampers RNAP-promoter escape (red region), and streptomycin, causing the formation of non-functional proteins (yellow region). It is assumed that cold shock mainly affects transcription, similarly to novobiocin [10].

First, we define a ‘detailed’ illustrative model based on the experiments conducted, specifically, cold shock, novobiocin, stationary growth-phase transition, rifampicin, and streptomycin (Fig 5). Shortly, the model assumes σ factor regulation (pink area, Fig 5) and a two rate-limiting-step process of transcription initiation: a step modelling promoter binding and finding of the transcription start site by RNAP holoenzymes, followed by a step modeling the processes that occur until promoter escape, such as the open-complex formation (blue area, Fig 5) [65,66]. Whereas all steps are reversible [66], we do not model reversibility, as we lack empirical data on *in vivo* parameter values for the events. These steps would increase noise in gene expression, but not mean rates.

Critically, the model assumes that the promoter can switch between two “transcription states” that differ in the rate of RNA production (blue area, Fig 5) [52]. This (and only this) allows one gene to have two distinct RNA production rates at different time points. For simplicity, we implement this difference in the rate of promoter binding by RNAP holoenzymes. However, in some genes with bimodal expression levels, it could be caused by different rates of promoter escape instead (or by differences in both rates). Note that many mechanisms could tune these rates, such as TFs. Here, we do not model explicitly the mechanisms making this possible.

Next, the model assumes that the promoter can be locked due to PSB and, thus, be influenced by the antibiotic novobiocin, as well as by cold shock, among other phenomena (green area, Fig 5) [10]. The effects of PSB and novobiocin can be set to differ with the promoter state. For simplicity, as a past study suggests that this is the step most influenced by cold shock, we do not model its influence on other steps [10].

The model also considers rifampicin. Separating transcription into two rate-limiting steps allows modelling that rifampicin specifically hampers promoter escape alone [55,56] (red area, Fig 5).

Meanwhile, translation, and the effects of streptomycin, are modeled by two separate (competing) sets of reactions. The first (translation, orange area), produces functional proteins. The second (yellow area), models the effect of streptomycin, namely the distortion of Ribosomes, which then produce nonfunctional proteins [57–59].

Finally, the model includes RNA and protein decay events (grey area). These decays account for degradation as well as dilution due to cell division.

**Fig 6:**
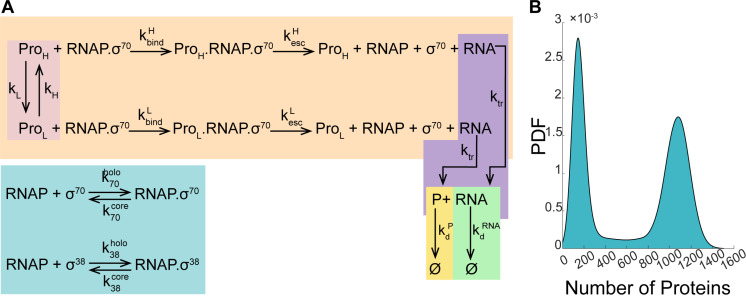
(A) Reduced stochastic model of bimodal gene expression. The model includes the transition between two states differing in transcription initiation rates, which generates bimodal single-cell proteins numbers. Also included is the reaction for RNA production in each state. Finally, there are reactions for RNA translation of RNAs as well as for RNA and protein degradation, which are not transcription state dependent. Perturbations are simplified by assuming that they change rate-constant values, rather than being modeled by independent reactions. All reactions, elements, and rate constants are described in detail in Section 1.7 in S1 Text. **(B)** Distribution of single-cell protein numbers resulting from simulations of the reduced stochastic model using the reference parameter values in Table B in S1 Text.

From the detailed model in Fig 5, we then developed a reduced stochastic model of bimodal gene expression. This is necessary, since we lack empirical data on several rate constants necessary to implement the detailed model. This model, shown in Fig 6A, also allows promoters to have two states (low ‘L’ and high ‘H’ transcription rates). Switching between states occurs at the rates given by constants *k*_*H*_ and *k*_*L*_, respectively. Transcription occurs at the corresponding rates constants, *k*_*bind*_^*H*^, **k*_*esc*_^*H*^*, *k*_*bind*_^*L*^ and *k*_*esc*_^*L*^. Once transcribed, RNAs are translated at a rate *k_tr_* independent from the promoter state. Finally, RNAs and proteins decay due to degradation and dilution due to cell division (*k*_*d*_^*RNA*^ or *k*_*d*_^*P*^), also independently from the promoter state.

Using this model, the effects of rifampicin are simulated by reducing the rates of promoter escape [55,56], *k*_*esc*_^*L*^ and *k*_*esc*_^*H*^, depending on the promoter state. Meanwhile, the effects of shifting to stationary growth phase are modeled by tuning the numbers of RNAP.σ^70^ and of RNAP.σ^38^ (for simplicity, we assume a promoter with σ^70^ preference). Also, to study the effects of different σ factor preferences, we tuned the rate of RNAP-promoter binding *k*_*bind*_^*L*^ or *k*_*bind*_^*H*^. Further, the effects of Novobiocin are modeled by changing *k*_*L*_ or *k*_*H*_, which tunes the expected time that a promoter is in each state [64], while the effects of streptomycin are modeled by reducing the rate of translation, *k*_*tr*_ [57]. Finally, we modeled the effects of changing decay rates of RNA and proteins, to simulate changes in cell division rates. In addition to this, one could model the effects of cold shock by tuning according to different degrees for all rates above (not done here as empirical data are not abundant).

From genome-wide measurements, we obtained reference values for each rate constant of the reduced model (Table B in S1 Text). The bimodal distribution of single-cell protein numbers obtained from simulations using these reference values is shown in Fig 6B. The distribution is bimodal, and exhibits a weak asymmetry, with a higher peak in the first mode.

It is noteworthy that the distribution produced by the model in Fig 6B has far less variability than any of the empirical distributions in Fig 4B. This is expected since the only source of variability in the model is the stochastic nature of the chemical reactions. Meanwhile, the sources of variability of the empirical distributions include not only the stochastic nature of the chemical reactions, but also several other sources, such as differences in cell components between individual cells, variability in the moments when the DNA replicates, variability in the asymmetries in partitioning of components during cell division, measurement uncertainties, among other.

Finally, we note that, in the model above, unimodal distributions emerge when the dynamics of two, distinct transcription events are sufficiently similar to make these events dynamically indistinguishable. For comparison, we next considered models that, instead, have the two-state system reduced into a single-state system. As such, these models’ reactions are the same as the reactions in Fig 6A (except that one of the two transcription reactions no longer exists). We considered three such models, which differ in the expression rate of the remaining transcription state, equaling: (i) the rate of the state (of the two original ones) with lower-expression, or (ii) the average of the rates of the two original states, or (iii) the rate of the original state with higher expression.

Fig P in S1 Text shows how the original bimodal distributions shift to unimodal in all three cases, after changing the transcription reactions of the system. All models take a similar time to exhibit unimodal single-cell distributions of protein numbers. Meanwhile, panel A of Fig Q in S1 Text shows that, as expected, these models differ in mean single-cell protein numbers, with only model (ii) matching the original model. For this, and more reasons, they also differ in the CV^2^ of single cell protein numbers (panel B of Fig Q in S1 Text).

Interestingly, for example, model (i) is in good agreement with the observed dynamics of *manX* and *paaX* under many of the perturbations (Fig 2). Meanwhile, model (ii) is in good agreement with the observed dynamics of, e.g., *metK* subjected to novobiocin (Fig 2). Finally, model (iii) describes well the dynamics of, e.g., *pgi* under cold shock.

### 2.8. Exploring the robustness of the bimodality using model simulations

**Fig 7:**
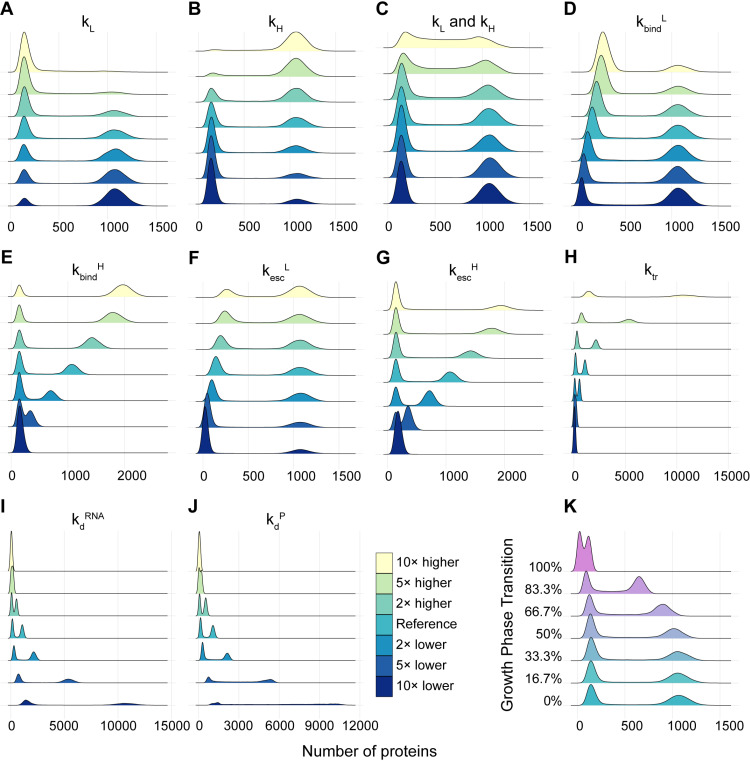
Single cell distributions of protein numbers estimated from simulations of the reduced model when tuning, respectively: **(A)** *k*_*L*_, **(B)** *k*_*H*_, **(C)** both *k*_*L*_ and *k*_*H*_ while keeping *k*_*H*_/*k*_*L*_constant **(D)** *k*_*bind*_^*L*^, **(E)** *k*_*bind*_^*H*^, **(F)** *k*_*esc*_^*L*^, **(G)** *k*_*esc*_^*H*^, **(H)** *k*_*tr*_, **(I)** *k*_*d*_^*RNA*^, **(J)** *k*_*d*_^*P*^, and **(K)** different number of σ^38^ and different rates of *k*_*tr*_, *k*_*d*_^*RNA*^, *k*_*d*_^*P*^ to mimic the gradual transition from exponential to stationary growth phases (0% and 100%, respectively).

We explored the reduced model by tuning, independently, each rate constant in Fig 6A, assuming that they can, at most, become 10× larger or 10x smaller than the reference value in Table B in S1 Text, respectively. This assumption is based on the empirical interquartile range of mean protein numbers reported in [24] (Fig R in S1 Text). The *in-silico* distributions of single-cell protein numbers are shown in Fig 7. For each bimodal distribution, let the ‘L peak’ be the peak corresponding to the lower expression rate and the ‘H peak’ be the peak corresponding to the higher expression rate.

Comparing the distributions qualitatively, first, changes in *k*_*d*_^*RNA*^ and *k*_*d*_^*P*^ cause the strongest increase in the distance between peaks and, at the same time, the strongest broadening of the modes. This relates with the influence of these rate constants on the noise in RNA and protein numbers, due to their direct control over the mean in RNA and protein numbers (as the mean numbers decrease, noise, as defined by CV^2^, should increase [67]).

Other parameters allowing large changes in the distance between the two modes are *k*_*bind*_^*H*^ and *k*_*esc*_^*H*^. This implies that the time that promoters remain occupied, i.e., unavailable for new RNAP bindings is critical in determining the distance between modes. On the other hand, *k*_*bind*_^*L*^ and *k*_*esc*_^*L*^ do not cause similar changes, albeit also controlling the time that promoters remain occupied. This relates to their smaller values relative to all other rate constants of the model (from Table B in S1 Text, *k*_*bind*_^*L*^ and *k*_*esc*_^*L*^ are one order of magnitude smaller than *k*_*bind*_^*H*^ and *k*_*esc*_^*H*^).

Finally, only a few conditions transform the distribution from bimodal into unimodal. Expectedly from above, only *k*_*bind*_^*H*^, *k*_*esc*_^*H*^ and *k*_*tr*_, at their lowest values, as well as *k*_*d*_^*RNA*^ and *k*_*d*_^*P*^ at their highest values achieve this transition. Contrarily, only when increasing both *k*_*L*_ as well as *k*_*H*_, does one observe a significant overlapping between the two modes of the distribution without loss of bimodality. This is in line with the robustness to perturbations of the bimodality of the genes observed above.

In addition to the simulations above, where we increased or decreased one rate constant at a time, we next considered a more complex transition. Namely, we modeled the events known to occur when cells shift to stationary growth. First, during this transition, the average lifetime of mRNAs increase to 7.8 min [68] while protein degradation rates increase by 8%/h [69]. Meanwhile, dilution due to cell division decreases by approximately 91.5% (as cells stop dividing). We model these changes by reducing decay rates *k*_*d*_^*RNA*^ and *k*_*d*_^*P*^ significantly. Additionally, translation activity also decreases 40 times when cells enter stationary phase [70]. Finally, there are changes in σ^38^ amounts, but also in the formation rate of RNAPs holoenzymes, due to changes in the levels of anti-sigma factors [71], 6S RNA [72–74], ppGpp/DksA [75], Crl [76], among others (this is approximately accounted for by changing the ratio of available holoenzymes, Section 1.7.3 in S1 Text).

We tuned the bimodal gene expression dynamics assuming gradual changes from the values in Table B in S1 Text to the values in Table C in S1 Text. Fig 7K shows gradual changes in the bimodal distribution of single cell protein numbers, with the mean proteins of the H and L peaks becoming smaller due to the reduction in the numbers of holoenzymes RNAP.σ^70^ and the increased decay rates of RNAs and proteins. Nevertheless, gene expression levels remain bimodal. This suggests that the genes measured whose distributions became unimodal in the stationary growth phase are subjected to even stronger changes than what is assumed in this model.

It is worth noting that, regarding the switching rates between the H and L states, at the moment, there is no empirical information on these rates. One could argue that, by setting these rates slower that all other rates (Table B in S1 Text), we made them unrealistically slow. We based ourselves on the simulations data. First, (i) in Fig 7C, if the transitions were faster than assumed, the two modes of the distribution would overlap (hampering bimodality). Similarly, (ii) the model shows (Fig 7I and 7J) that if the degradation rates become fast relative to the switching frequencies, again the distribution shifts to unimodal. As such, we expect the switching frequencies to be slower than protein degradation rates in *E. coli* (which are in the order of tens of minutes).

Finally, observing Fig 7, note that in all cases where the bimodal distributions shifted to unimodal by tuning the parameter values, the unimodal distributions are centered at the original ‘low’ state. This occurred because of the boundaries that were set for the parameter values. To show this, we expanded this range for the parameters *k*_*bind*_^*L*^ and *k*_*esc*_^*L*^ (Fig 7D and 7F, respectively). The results, in Fig S in S1 Text, show that, for these wider ranges, the bimodal distribution shifts to unimodal distributions centered around the original ‘high’ state, as long as the other rate-limiting step is increased to match the value in the ‘high’ state.

### 2.9. Potential sources of bimodality

We first investigated potential common sources of bimodality of the 7 genes. However, since the bimodality is observed also from the genes’ promoters when on plasmids, we excluded the accumulation of positive supercoiling or RNAP collisions due to the activity of neighboring genes as the sources of bimodality. Similarly, we excluded RNA silencing or sequences enhancing transcriptional pausing, since the plasmids only code for the promoter regions of the genes of interest followed by the coding region of the fluorescent protein (i.e., they do not code for the original RNA of the gene of interest or for any membrane-associated protein). Further, we did not find any known TF regulator common to any two genes with bimodal expression levels here reported.

While we did not find a common explanation for all genes, we did find potential, diverse explanations for each but all genes. First, the bimodality could emerge from the regulation by each gene’s specific input TF(s), including a self-regulator TF. This is the case for the *paaX* gene, who is capable of autorepression [46]. This can lead to bimodality if the repression is nonlinear due to, e.g., feedback delays [77] or if the repression has a switch-like behavior.

While we did not find any other evidence of self-regulation, positive feedback loops are expected to be able of generating bimodality as well. To test, we modeled a positive feedback loop (Fig T in S1 Text) whose parameter values are largely based on the reduced model. From simulations (Fig T in S1 Text), using these values one observes bimodality. Moreover, akin to the reduced bimodal model, altering the transcription rate-limiting steps (RNAP binding and promoter escape rates) changes the distance between the peaks. Meanwhile, changing the ratio of binding and unbinding of the proteins to the promoter region (i.e., the rates of gene activation), changes the height of the peaks (relative to each other), similarly to when changing the rates of shifting between the low and high states in the reduced model.

Another source of bimodality could be TFs with ‘dual effects’. This may occur in the cases of *elaB* and *lldD* genes (OxyR and LldR TFs, respectively) [46]. Their TF regulators can switch between being activating or repressing based on environmental or intracellular conditions [78]. Slight differences in these signals among individual cells can drive some cells will have the TF acting as an activator, while others will have the TF acting as a repressor. This can lead to bimodal gene expression within a cell population by enabling the coexistence of two distinct subpopulations: one with high expression and the other with low expression.

Another potential mechanism generating bimodality is multiple σ factor preference. This is the case of *pgi* and *metK* genes, as their promoters can be recognize by σ^70^ and by σ^38^ [46]. This could lead to bimodal distributions if the transcription rates differ with the σ factor [65]. In these genes, cell-to-cell variability in σ factors numbers could potentially cause significant cell-to-cell variability in transcription rates, thus creating bimodal distributions of gene expression.

Finally, *manX* is regulated by nine TFs, including two activators and two repressors. Such a complex regulatory mechanism could be subject to significant cell-to-cell variability, particularly if small differences in the numbers of the nine transcription factors can have cumulative effects.

Finally, we were unable to identify any potential regulatory mechanism that could explain the bimodal expression observed in the *pyrH* gene. Given these regulatory differences, the cause of bimodal expression in these genes remains unclear, suggesting that other factors may be contributing to the observed expression patterns.

## 3. Discussion

The ability of genes to be multi-stable, i.e., to stably express at distinct rates under a given condition, is an essential, evolved survival strategy, that allows genetically identical organisms to exhibit different phenotypes, enhancing their adaptability, particularly under unpredictable environments [4,79–82]. Recognizing genes with these properties remains challenging, making it even more challenging to study bimodality, such as its robustness to stress and responsiveness to external signals, which are critical properties, if they are to be used as building blocks of synthetic circuits.

We found seven *E. coli* genes exhibiting bimodality under standard growth conditions and studied the robustness of their bimodality to changes in σ factor regulation, temperature shifts, and antibiotics targeting transcription, translation, and supercoiling curation, respectively. We observed that, surprisingly, these seven genes alone already occupy a very wide region of the state space of mean versus noise in single cell protein numbers. This suggests that bimodality can emerge within a wide range of gene regulatory conditions, rather than being limited to a very narrow set of properties. This also suggests that modality might be significantly robust to a wide range of conditions.

Regarding the stress response of the seven genes, we also observed a wide range of behaviors. All genes exhibited stress-specific changes in their bimodal distributions and most of the genes lost bimodality when subject to at least one, or more, of the stresses studied. However, at least one or more of the genes were robust to each of the stresses tested. This entails that, taken together as a set of future building blocks of synthetic circuits, the strains coding for these genes already form a library that can be of use in a wide range of conditions, having both robustness as well as responsiveness to the stresses.

Another fundamental property observed, particularly if these genes are to be used as a strain library, was reversibility. Namely, after observing the circuits losing bimodality under stationary growth conditions, we next observed the recovery of such bimodality when the cells were once again provided fresh media. We believe that reversibility will also occur in the case of the other stresses studied, e.g., when the antibiotics dilute sufficiently with cell division or when returning cells from cold to optimal temperature conditions.

In the future, in addition to finding more genes with bimodal behaviors, first, it would be important to study in detail the reversibility of the bimodality of these genes to stresses other than the shift to stationary growth conditions. Secondly, if we are to use these genes in synthetic circuits, another necessary step will be dissecting what regulatory mechanisms provide them with bimodality. Such knowledge is particularly necessary for being able to externally control them. Since many potential mechanisms may make their bimodality possible [34], this search could be cumbersome, particularly since the mechanism may differ between the seven genes. Moreover, potentially, in some of the genes, the bimodality might result from the combined effect of two or more mechanisms. However, our results suggest that these mechanisms act on transcription initiation (between RNAP binding and promoter escape, during which closed and open complex formations occur), which reduces the search space significantly. Thirdly, some genes, in addition to reversibility, also exhibit hysteresis under certain stresses. Future studies could be performed to determine how the regulatory mechanism of these genes could be used as memory units of future synthetic genetic circuits [83].

A fourth important study would be one to determine what is the role of these seven genes (and of other genes with bimodality) in the selection of transcriptional programs of stress response involving hundreds of genes. For example, these genes may be of particular importance in programs related to antibiotic persistence and tolerance, where some cells of a population opt for behaving differently from most other cells, when under antibiotic stress.

Moreover, research should be conducted on whether bimodal gene expression is common in other bacterial species and what biofunctions genes with bimodal expression are related with. Potentially, the findings might be of use in cancer research, where gene expression bimodality has been suggested to play a significant role [84–88].

Meanwhile, the model proposed here was found to mimic the dynamics with realism, including the stress responses. As noted, the model identified *k*_*d*_^*RNA*^, *k*_*d*_^*P*^, *k*_*bind*_^*H*^*,* and *k*_*esc*_^*H*^ has the rate constants that, when tuned, most influence the dynamics. Interestingly, from a synthetic biology point of view, while the first two rate constants are not easily tunable, the latter two rates are, as they differ significantly between promoters (natural and synthetic). This might allow engineering circuits with diverse dynamics, by placing the promoters with more appropriate dynamics for the aims set. Importantly, such dynamics can be known prior to this, e.g., using existing strain libraries [24,63,89]. In the future, this model could be used to explore how, e.g., antibiotics alter the bimodality using realistic parameter values, in *E. coli* as well as in other bacteria, and identify the influential parameters making this possible. It can also be used to predict the effects of other perturbations, and, if tuned accordingly, assist the design of synthetic circuits in other bacteria as well.

Finally, potentially, as mentioned above, the most important, easily executable, outcome of this study would be to use the genes identified as bimodal to engineer a new strain library, with each strain carrying a plasmid coding for a promoter with bimodal dynamics controlling the expression of RNA coding for a fluorescent protein along with multiple restriction sites to allow the insertion of sequences of interest coding for TFs, other proteins, siRNAs, additional fluorescent proteins, etc. This library would be of immediate use, e.g., in transforming any circuit of interest into bimodal. This could be of interest for bioindustrial applications, by enhancing the plasticity of synthetic circuits.

## 4. Materials and methods

To produce the empirical data, we used *E. coli*’s YFP [24] (Table E in S1 Text) and GFP promoter fusion ([63] libraries (Section 1.1 in S1 Text). Single cell gene expression measurements conducted by flow cytometry and cells preparation are described in (Section 1.2 in S1 Text). Additional measurements by microscopy and corresponding image analysis are described in (Section 1.3 in S1 Text). Distributions were classified as unimodal or bimodal using the methods described in (Section 1.4 in S1 Text). Finally, linear fits of the empirical data using regression models is described in (Section 1.5 in S1 Text).

To produce *in silico* data from the models, we performed simulations as described in (Section 1.6 in S1 Text). The values of all rate constants of the model were estimated from empirical data, as described in Section 1.7 in S1 Text.

A data package with flow cytometry and microscopy data was deposited in Dryad under the https://doi.org/10.5061/dryad.dz08kps5n [90].

## Supporting information

S1 Text Supplementary information. Supplementary methods 1.1–1.7, supplementary figures A–V and supplementary tables A–E.
(DOCX)

## References

[1] Blount ZD, Maddamsetti R, Grant NA, Ahmed ST, Jagdish T, Baxter JA, et al. Genomic and phenotypic evolution of Escherichia coli in a novel citrate-only resource environment. Elife. 2020;9:e55414. doi: 10.7554/eLife.55414 32469311 PMC7299349

[2] Finkel SE. Long-term survival during stationary phase: evolution and the GASP phenotype. Nat Rev Microbiol. 2006;4(2):113–20. doi: 10.1038/nrmicro1340 16415927

[3] Balaban NQ, Merrin J, Chait R, Kowalik L, Leibler S. Bacterial persistence as a phenotypic switch. Science. 2004;305(5690):1622–5. doi: 10.1126/science.1099390 15308767

[4] Acar M, Mettetal JT, van Oudenaarden A. Stochastic switching as a survival strategy in fluctuating environments. Nat Genet. 2008;40(4):471–5. doi: 10.1038/ng.110 18362885

[5] Phadtare S, Inouye M. Genome-wide transcriptional analysis of the cold shock response in wild-type and cold-sensitive, quadruple-csp-deletion strains of Escherichia coli. J Bacteriol. 2004;186(20):7007–14. doi: 10.1128/JB.186.20.7007-7014.2004 15466053 PMC522181

[6] Jozefczuk S, Klie S, Catchpole G, Szymanski J, Cuadros-Inostroza A, Steinhauser D, et al. Metabolomic and transcriptomic stress response of Escherichia coli. Mol Syst Biol. 2010;6:364. doi: 10.1038/msb.2010.18 20461071 PMC2890322

[7] Fang X, Sastry A, Mih N, Kim D, Tan J, Yurkovich JT, et al. Global transcriptional regulatory network for Escherichia coli robustly connects gene expression to transcription factor activities. Proc Natl Acad Sci U S A. 2017;114(38):10286–91. doi: 10.1073/pnas.1702581114 28874552 PMC5617254

[8] Kochanowski K, Gerosa L, Brunner SF, Christodoulou D, Nikolaev YV, Sauer U. Few regulatory metabolites coordinate expression of central metabolic genes in Escherichia coli. Mol Syst Biol. 2017;13(1):903. doi: 10.15252/msb.20167402 28049137 PMC5293157

[9] Almeida BLB, Bahrudeen MN, Chauhan V, Dash S, Kandavalli V, Häkkinen A, et al. The transcription factor network of E. coli steers global responses to shifts in RNAP concentration. Nucleic Acids Res. 2022;50(12):6801–19. doi: 10.1093/nar/gkac540 35748858 PMC9262627

[10] Dash S, Palma CSD, Baptista ISC, Almeida BLB, Bahrudeen MNM, Chauhan V, et al. Alteration of DNA supercoiling serves as a trigger of short-term cold shock repressed genes of E. coli. Nucleic Acids Res. 2022;50(15):8512–28. doi: 10.1093/nar/gkac643 35920318 PMC9410904

[11] Kussell E, Leibler S. Phenotypic diversity, population growth, and information in fluctuating environments. Science. 2005;309(5743):2075–8. doi: 10.1126/science.1114383 16123265

[12] Garcia-Bernardo J, Dunlop MJ. Phenotypic diversity using bimodal and unimodal expression of stress response proteins. Biophys J. 2016;110(10):2278–87. doi: 10.1016/j.bpj.2016.04.012 27224492 PMC4881231

[13] Reyes Ruiz LM, Williams CL, Tamayo R. Enhancing bacterial survival through phenotypic heterogeneity. PLoS Pathog. 2020;16(5):e1008439. doi: 10.1371/journal.ppat.1008439 32437427 PMC7241687

[14] Kussell E, Kishony R, Balaban NQ, Leibler S. Bacterial persistence: a model of survival in changing environments. Genetics. 2005;169(4):1807–14. doi: 10.1534/genetics.104.035352 15687275 PMC1449587

[15] Lachmann M, Jablonka E. The inheritance of phenotypes: an adaptation to fluctuating environments. J Theor Biol. 1996;181(1):1–9. doi: 10.1006/jtbi.1996.0109 8796186

[16] Thattai M, van Oudenaarden A. Stochastic gene expression in fluctuating environments. Genetics. 2004;167(1):523–30. doi: 10.1534/genetics.167.1.523 15166174 PMC1470854

[17] Wolf DM, Vazirani VV, Arkin AP. Diversity in times of adversity: probabilistic strategies in microbial survival games. J Theor Biol. 2005;234(2):227–53. doi: 10.1016/j.jtbi.2004.11.020 15757681

[18] Dubnau D, Losick R. Bistability in bacteria. Mol Microbiol. 2006;61(3):564–72. doi: 10.1111/j.1365-2958.2006.05249.x 16879639

[19] Avery SV. Microbial cell individuality and the underlying sources of heterogeneity. Nat Rev Microbiol. 2006;4(8):577–87. doi: 10.1038/nrmicro1460 16845428

[20] Siegele DA, Hu JC. Gene expression from plasmids containing the *araBAD* promoter at subsaturating inducer concentrations represents mixed populations. Proc Natl Acad Sci U S A. 1997;94(15):8168–72. doi: 10.1073/pnas.94.15.8168 9223333 PMC21575

[21] Koirala S, Mears P, Sim M, Golding I, Chemla YR, Aldridge PD, et al. A nutrient-tunable bistable switch controls motility in Salmonella enterica serovar Typhimurium. mBio. 2014;5(5):e01611–14. doi: 10.1128/mBio.01611-14 25161191 PMC4173784

[22] Rattray JB, Thomas SA, Wang Y, Molotkova E, Gurney J, Varga JJ, et al. Bacterial quorum sensing allows graded and bimodal cellular responses to variations in population density. mBio. 2022;13(3):e0074522. doi: 10.1128/mbio.00745-22 35583321 PMC9239169

[23] Leh H, Khodr A, Bouger M-C, Sclavi B, Rimsky S, Bury-Moné S. Bacterial-chromatin structural proteins regulate the bimodal expression of the locus of enterocyte effacement (LEE) pathogenicity island in enteropathogenic Escherichia coli. mBio. 2017;8(4):e00773–17. doi: 10.1128/mBio.00773-17 28790204 PMC5550750

[24] Taniguchi Y, Choi PJ, Li G-W, Chen H, Babu M, Hearn J, et al. Quantifying E. coli proteome and transcriptome with single-molecule sensitivity in single cells. Science. 2010;329(5991):533–8. doi: 10.1126/science.1188308 20671182 PMC2922915

[25] Gamba P, Jonker MJ, Hamoen LW. A novel feedback loop that controls bimodal expression of genetic competence. PLoS Genet. 2015;11(6):e1005047. doi: 10.1371/journal.pgen.1005047 26110430 PMC4482431

[26] Lamprecht O, Ratnikava M, Jacek P, Kaganovitch E, Buettner N, Fritz K, et al. Regulation by cyclic di-GMP attenuates dynamics and enhances robustness of bimodal curli gene activation in Escherichia coli. PLoS Genet. 2023;19(5):e1010750. doi: 10.1371/journal.pgen.1010750 37186613 PMC10212085

[27] Charlebois DA, Hauser K, Marshall S, Balázsi G. Multiscale effects of heating and cooling on genes and gene networks. Proc Natl Acad Sci U S A. 2018;115(45):E10797–806. doi: 10.1073/pnas.1810858115 30341217 PMC6233105

[28] Pelet S, Rudolf F, Nadal-Ribelles M, de Nadal E, Posas F, Peter M. Transient activation of the HOG MAPK pathway regulates bimodal gene expression. Science. 2011;332(6030):732–5. doi: 10.1126/science.1198851 21551064

[29] Wilhelm T. The smallest chemical reaction system with bistability. BMC Syst Biol. 2009;3:90. doi: 10.1186/1752-0509-3-90 19737387 PMC2749052

[30] Ochab-Marcinek A, Tabaka M. Bimodal gene expression in noncooperative regulatory systems. Proc Natl Acad Sci U S A. 2010;107(51):22096–101. doi: 10.1073/pnas.1008965107 21135209 PMC3009792

[31] Jia C, Grima R. Dynamical phase diagram of an auto-regulating gene in fast switching conditions. J Chem Phys. 2020;152(17):174110. doi: 10.1063/5.0007221 32384856

[32] Jia C, Li Y. Analytical time-dependent distributions for gene expression models with complex promoter switching mechanisms. SIAM J Appl Math. 2023;83(4):1572–602. doi: 10.1137/22m147219x

[33] Cao Z, Filatova T, Oyarzún DA, Grima R. A stochastic model of gene expression with polymerase recruitment and pause release. Biophys J. 2020;119(5):1002–14. doi: 10.1016/j.bpj.2020.07.020 32814062 PMC7474183

[34] Ferrell JE Jr. Self-perpetuating states in signal transduction: positive feedback, double-negative feedback and bistability. Curr Opin Cell Biol. 2002;14(2):140–8. doi: 10.1016/s0955-0674(02)00314-9 11891111

[35] Hasty J, McMillen D, Isaacs F, Collins JJ. Computational studies of gene regulatory networks: in numero molecular biology. Nat Rev Genet. 2001;2(4):268–79. doi: 10.1038/35066056 11283699

[36] Scandolo CM, Gour G, Sanders BC. Covariant influences for finite discrete dynamical systems. Phys Rev E. 2023;107(1):014203. doi: 10.1103/PhysRevE.107.014203 36797937

[37] Baptista ISC, Kandavalli V, Chauhan V, Bahrudeen MNM, Almeida BLB, Palma CSD, et al. Sequence-dependent model of genes with dual σ factor preference. Biochim Biophys Acta Gene Regul Mech. 2022;1865(3):194812. doi: 10.1016/j.bbagrm.2022.194812 35338024

[38] Cunningham A. Fluorescence pulse shape as a morphological indicator in the analysis of colonial microalgae by flow cytometry. J Microbiol Methods. 1990;11(1):27–36. doi: 10.1016/0167-7012(90)90044-7

[39] Gross CA, Chan C, Dombroski A, Gruber T, Sharp M, Tupy J, et al. The functional and regulatory roles of sigma factors in transcription. Cold Spring Harb Symp Quant Biol. 1998;63:141–55. doi: 10.1101/sqb.1998.63.141 10384278

[40] Helmann JD, Chamberlin MJ. Structure and function of bacterial sigma factors. Annu Rev Biochem. 1988;57:839–72. doi: 10.1146/annurev.bi.57.070188.004203 3052291

[41] Weber H, Polen T, Heuveling J, Wendisch VF, Hengge R. Genome-wide analysis of the general stress response network in Escherichia coli: sigmaS-dependent genes, promoters, and sigma factor selectivity. J Bacteriol. 2005;187(5):1591–603. doi: 10.1128/JB.187.5.1591-1603.2005 15716429 PMC1063999

[42] Hengge R. Proteolysis of sigmaS (RpoS) and the general stress response in Escherichia coli. Res Microbiol. 2009;160(9):667–76. doi: 10.1016/j.resmic.2009.08.014 19765651

[43] Farewell A, Kvint K, Nyström T. Negative regulation by RpoS: a case of sigma factor competition. Mol Microbiol. 1998;29(4):1039–51. doi: 10.1046/j.1365-2958.1998.00990.x 9767572

[44] Kandavalli VK, Tran H, Ribeiro AS. Effects of σ factor competition are promoter initiation kinetics dependent. Biochim Biophys Acta. 2016;1859(10):1281–8. doi: 10.1016/j.bbagrm.2016.07.011 27452766

[45] Mauri M, Klumpp S. A model for sigma factor competition in bacterial cells. PLoS Comput Biol. 2014;10(10):e1003845. doi: 10.1371/journal.pcbi.1003845 25299042 PMC4191881

[46] Salgado H, Gama-Castro S, Lara P, Mejia-Almonte C, Alarcón-Carranza G, López-Almazo AG, et al. RegulonDB v12.0: a comprehensive resource of transcriptional regulation in E. coli K-12. Nucleic Acids Res. 2024;52(D1):D255–64. doi: 10.1093/nar/gkad1072 37971353 PMC10767902

[47] Kakeda M, Ueguchi C, Yamada H, Mizuno T. An Escherichia coli curved DNA-binding protein whose expression is affected by the stationary phase-specific sigma factor sigma S. Mol Gen Genet. 1995;248(5):629–34. doi: 10.1007/BF02423459 7476863

[48] Gellert M, O’Dea MH, Itoh T, Tomizawa J. Novobiocin and coumermycin inhibit DNA supercoiling catalyzed by DNA gyrase. Proc Natl Acad Sci U S A. 1976;73(12):4474–8. doi: 10.1073/pnas.73.12.4474 794878 PMC431506

[49] Knox C, Wilson M, Klinger CM, Franklin M, Oler E, Wilson A, et al. DrugBank 6.0: the DrugBank Knowledgebase for 2024. Nucleic Acids Res. 2024;52(D1):D1265–75. doi: 10.1093/nar/gkad976 37953279 PMC10767804

[50] Oliveira SMD, Häkkinen A, Lloyd-Price J, Tran H, Kandavalli V, Ribeiro AS. Temperature-dependent model of multi-step transcription initiation in Escherichia coli based on live single-cell measurements. PLoS Comput Biol. 2016;12(10):e1005174. doi: 10.1371/journal.pcbi.1005174 27792724 PMC5085040

[51] Polissi A, De Laurentis W, Zangrossi S, Briani F, Longhi V, Pesole G, et al. Changes in Escherichia coli transcriptome during acclimatization at low temperature. Res Microbiol. 2003;154(8):573–80. doi: 10.1016/S0923-2508(03)00167-0 14527658

[52] McClure WR. Mechanism and control of transcription initiation in prokaryotes. Annu Rev Biochem. 1985;54:171–204. doi: 10.1146/annurev.bi.54.070185.001131 3896120

[53] Oliveira SMD, Neeli-Venkata R, Goncalves NSM, Santinha JA, Martins L, Tran H, et al. Increased cytoplasm viscosity hampers aggregate polar segregation in Escherichia coli. Mol Microbiol. 2016;99(4):686–99. doi: 10.1111/mmi.13257 26507787

[54] Hartkoorn RC, Sala C, Magnet SJ, Chen JM, Pojer F, Cole ST. Sigma factor F does not prevent rifampin inhibition of RNA polymerase or cause rifampin tolerance in Mycobacterium tuberculosis. J Bacteriol. 2010;192(20):5472–9. doi: 10.1128/JB.00687-10 20729364 PMC2950495

[55] Campbell EA, Pavlova O, Zenkin N, Leon F, Irschik H, Jansen R, et al. Structural, functional, and genetic analysis of sorangicin inhibition of bacterial RNA polymerase. EMBO J. 2005;24(4):674–82. doi: 10.1038/sj.emboj.7600499 15692574 PMC549610

[56] McClure WR, Cech CL. On the mechanism of rifampicin inhibition of RNA synthesis. J Biol Chem. 1978;253(24):8949–56. doi: 10.1016/s0021-9258(17)34269-2 363713

[57] Luzzatto L, Apirion D, Schlessinger D. Mechanism of action of streptomycin in E. coli: interruption of the ribosome cycle at the initiation of protein synthesis. Proc Natl Acad Sci U S A. 1968;60(3):873–80. doi: 10.1073/pnas.60.3.873 4875806 PMC225133

[58] Demirci H, Murphy F 4th, Murphy E, Gregory ST, Dahlberg AE, Jogl G. A structural basis for streptomycin-induced misreading of the genetic code. Nat Commun. 2013;4:1355. doi: 10.1038/ncomms2346 23322043 PMC3552334

[59] Drummond DA, Wilke CO. The evolutionary consequences of erroneous protein synthesis. Nat Rev Genet. 2009;10(10):715–24. doi: 10.1038/nrg2662 19763154 PMC2764353

[60] Balakrishnan R, Mori M, Segota I, Zhang Z, Aebersold R, Ludwig C, et al. Principles of gene regulation quantitatively connect DNA to RNA and proteins in bacteria. Science. 2022;378(6624):eabk2066. doi: 10.1126/science.abk2066 36480614 PMC9804519

[61] Veening J-W, Smits WK, Kuipers OP. Bistability, epigenetics, and bet-hedging in bacteria. Annu Rev Microbiol. 2008;62:193–210. doi: 10.1146/annurev.micro.62.081307.163002 18537474

[62] Ozbudak EM, Thattai M, Lim HN, Shraiman BI, Van Oudenaarden A. Multistability in the lactose utilization network of Escherichia coli. Nature. 2004;427(6976):737–40. doi: 10.1038/nature02298 14973486

[63] Zaslaver A, Bren A, Ronen M, Itzkovitz S, Kikoin I, Shavit S, et al. A comprehensive library of fluorescent transcriptional reporters for Escherichia coli. Nat Methods. 2006;3(8):623–8. doi: 10.1038/nmeth895 16862137

[64] Chong S, Chen C, Ge H, Xie XS. Mechanism of transcriptional bursting in bacteria. Cell. 2014;158(2):314–26. doi: 10.1016/j.cell.2014.05.038 25036631 PMC4105854

[65] Browning DF, Busby SJ. The regulation of bacterial transcription initiation. Nat Rev Microbiol. 2004;2(1):57–65. doi: 10.1038/nrmicro787 15035009

[66] deHaseth PL, Zupancic ML, Record MT Jr. RNA polymerase-promoter interactions: the comings and goings of RNA polymerase. J Bacteriol. 1998;180(12):3019–25. doi: 10.1128/JB.180.12.3019-3025.1998 9620948 PMC107799

[67] Paulsson J. Models of stochastic gene expression. Phys Life Rev. 2005;2(2):157–75. doi: 10.1016/j.plrev.2005.03.003

[68] Chen H, Shiroguchi K, Ge H, Xie XS. Genome-wide study of mRNA degradation and transcript elongation in Escherichia coli. Mol Syst Biol. 2015;11(1):781. doi: 10.15252/msb.20145794 25583150 PMC4332155

[69] Maurizi MR. Proteases and protein degradation in Escherichia coli. Experientia. 1992;48(2):178–201. doi: 10.1007/BF01923511 1740190

[70] Reier K, Liiv A, Remme J. Ribosome protein composition mediates translation during the Escherichia coli stationary phase. Int J Mol Sci. 2023;24(4):3128. doi: 10.3390/ijms24043128 36834540 PMC9959377

[71] Hughes KT, Mathee K. The anti-sigma factors. Annu Rev Microbiol. 1998;52:231–86. doi: 10.1146/annurev.micro.52.1.231 9891799

[72] Wassarman KM. 6S RNA: a small RNA regulator of transcription. Curr Opin Microbiol. 2007;10(2):164–8. doi: 10.1016/j.mib.2007.03.008 17383220

[73] Sharma UK, Chatterji D. Transcriptional switching in Escherichia coli during stress and starvation by modulation of sigma activity. FEMS Microbiol Rev. 2010;34(5):646–57. doi: 10.1111/j.1574-6976.2010.00223.x 20491934

[74] Gildehaus N, Neusser T, Wurm R, Wagner R. Studies on the function of the riboregulator 6S RNA from E. coli: RNA polymerase binding, inhibition of in vitro transcription and synthesis of RNA-directed de novo transcripts. Nucleic Acids Res. 2007;35(6):1885–96. doi: 10.1093/nar/gkm085 17332013 PMC1874619

[75] Dalebroux ZD, Swanson MS. ppGpp: magic beyond RNA polymerase. Nat Rev Microbiol. 2012;10(3):203–12. doi: 10.1038/nrmicro2720 22337166 PMC13198741

[76] England P, Westblade LF, Karimova G, Robbe-Saule V, Norel F, Kolb A. Binding of the unorthodox transcription activator, Crl, to the components of the transcription machinery. J Biol Chem. 2008;283(48):33455–64. doi: 10.1074/jbc.M807380200 18818199 PMC2586269

[77] Zavala E, Marquez-Lago TT. Delays induce novel stochastic effects in negative feedback gene circuits. Biophys J. 2014;106(2):467–78. doi: 10.1016/j.bpj.2013.12.010 24461022 PMC3907244

[78] Ma J. Crossing the line between activation and repression. Trends Genet. 2005;21(1):54–9. doi: 10.1016/j.tig.2004.11.004 15680515

[79] Cağatay T, Turcotte M, Elowitz MB, Garcia-Ojalvo J, Süel GM. Architecture-dependent noise discriminates functionally analogous differentiation circuits. Cell. 2009;139(3):512–22. doi: 10.1016/j.cell.2009.07.046 19853288

[80] Kaern M, Elston TC, Blake WJ, Collins JJ. Stochasticity in gene expression: from theories to phenotypes. Nat Rev Genet. 2005;6(6):451–64. doi: 10.1038/nrg1615 15883588

[81] Elowitz MB, Leibler S. A synthetic oscillatory network of transcriptional regulators. Nature. 2000;403(6767):335–8. doi: 10.1038/35002125 10659856

[82] Ribeiro AS. Dynamics and evolution of stochastic bistable gene networks with sensing in fluctuating environments. Phys Rev E Stat Nonlin Soft Matter Phys. 2008;78(6 Pt 1):061902. doi: 10.1103/PhysRevE.78.061902 19256863

[83] Thomas P, Popović N, Grima R. Phenotypic switching in gene regulatory networks. Proc Natl Acad Sci U S A. 2014;111(19):6994–9. doi: 10.1073/pnas.1400049111 24782538 PMC4024914

[84] Moody L, Xu GB, Pan Y-X, Chen H. Genome-wide cross-cancer analysis illustrates the critical role of bimodal miRNA in patient survival and drug responses to PI3K inhibitors. PLoS Comput Biol. 2022;18(5):e1010109. doi: 10.1371/journal.pcbi.1010109 35639779 PMC9187341

[85] Hellwig B, Hengstler JG, Schmidt M, Gehrmann MC, Schormann W, Rahnenführer J. Comparison of scores for bimodality of gene expression distributions and genome-wide evaluation of the prognostic relevance of high-scoring genes. BMC Bioinformatics. 2010;11:276. doi: 10.1186/1471-2105-11-276 20500820 PMC2892466

[86] Wu M, Liu L, Chan C. Identification of novel targets for breast cancer by exploring gene switches on a genome scale. BMC Genomics. 2011;12:547. doi: 10.1186/1471-2164-12-547 22053771 PMC3269833

[87] Wolf DM, Lenburg ME, Yau C, Boudreau A, van ’t Veer LJ. Gene co-expression modules as clinically relevant hallmarks of breast cancer diversity. PLoS ONE. 2014;9(2):e88309. doi: 10.1371/journal.pone.0088309 24516633 PMC3917875

[88] Teschendorff AE, Miremadi A, Pinder SE, Ellis IO, Caldas C. An immune response gene expression module identifies a good prognosis subtype in estrogen receptor negative breast cancer. Genome Biol. 2007;8(8):R157. doi: 10.1186/gb-2007-8-8-r157 17683518 PMC2374988

[89] Dash S, Jagadeesan R, Baptista ISC, Chauhan V, Kandavalli V, Oliveira SMD, et al. A library of reporters of the global regulators of gene expression in Escherichia coli. mSystems. 2024;9(6):e0006524. doi: 10.1128/msystems.00065-24 38687030 PMC11237500

[90] BaptistaI, DashS, ArshA, KandavalliV, ScandoloCM, SandersB, et al. Bimodality in E. coli gene expression: sources and robustness to genome-wide stresses [Dataset]. Dryad. 2025. doi: 10.5061/dryad.dz08kps5n39946496

