# Supplementary material for: Bimodality in E. coli gene expression: Sources and robustness to genome-wide stresses
Source: PLoS Comput Biol. 2025 Feb 13;21(2):e1012817. doi: 10.1371/journal.pcbi.1012817 (PMC11825099; doi:10.1371/journal.pcbi.1012817)
Supplement: S1 Text — Supplementary methods 1.1–1.7, supplementary figures A–V and supplementary tables A–E. (DOCX) [file pcbi.1012817.s001.docx]

**Supplementary material for** **“Bimodality in *E. coli* gene expression: sources and robustness to genome-wide stresses”**

**Supplementary Methods**

- 1. **Strains**

*E. coli* strains are listed in Table E in S1 Text. We used YFP strains from the genetic stock center (CGSC) of Yale University, U.S.A [1]. The expression of fluorescent proteins is under the control of the genes of interest. Based on the results in [1], we refer to cell fluorescence levels as protein levels. We also used GFP transcriptional fusions of *E. coli* promoters to measure promoter activity [2].

- 1. **Single-cell, gene expression measurements**

From a glycerol stock (-80 °C), cells were streaked on LB agar plates (2%) and incubated at 37 °C, overnight. A single colony was picked, inoculated in LB medium with antibiotics, and incubated at 30°C overnight with shaking. Overnight cultures were further diluted into fresh medium to an optical density of 600 nm (O.D._600_) of 0.03 and incubated for growth by shaking at 250 rpm at 37°C. O.D._600_ was recorded every 20 min for 1200 min. Cells were extracted at 150 min after inoculation into fresh M9 medium (1xM9 Salts, 2 mM MgSO_4_, 0.1 mM CaCl_2_; 5xM9 Salts 34 g/L Na_2_HPO_4_, 15 g/L KH_2_PO_4_, 2.5 g/L NaCl, 5 g/L NH_4_Cl) supplemented with 0.2% [Casamino acids](https://www.sciencedirect.com/topics/biochemistry-genetics-and-molecular-biology/casamino-acid) and 0.4% glucose to represent cells in exponential growth phase (Fig U in S1 Text).

To measure gene expression, we performed flow cytometry using an ACEA NovoCyte Flow Cytometer controlled by Novo Express V1.50. For this, cells were diluted 1:10000 into 1 ml of PBS (1X) vortexed for 10 sec. For each gene and condition, we performed 3 biological replicates, acquiring 50,000 events for each. We used the blue laser (488 nm) for excitation and the FITC-H channel (520/20 nm ﬁlter) for emission. We collected events at a flow rate of 14 µL/minute, a core diameter of 7.7 µM, and PMT voltage of 600. We set lower bound for the detection threshold in FSC-H was set to 5000 to remove interference from particles. We also removed the “far-out” outliers from FITC-H distributions of using Tukey fences [3]. Background subtraction was not performed to avoid altering the shape of the distributions.

To test the tunability of bimodal genes, we subjected the cells to different stresses targeting individual steps of transcription, upon reaching the O.D._600_ of 0.3. Specifically, cold shock was imposed by placing cells at 15 °C, monitored using a thermometer. For DNA gyrase and RNAP inhibition, we added novobiocin (50 μg/mL) and rifampicin (2.5 μg/mL) respectively. Similarly, for inhibiting translation initiation, antibiotic streptomycin (10 µg/mL) was added to the cells. The gene expression measurement timings were set to ~120 minutes after the perturbations to allow for post transcriptional changes occurred [4]. Additionally, these timings ensure that the changes observed derived mainly from the effects on the regulation mechanisms, rather than due to indirect causes such as signal propagation in the TF network (visible at ~180 minutes after the perturbation) [5].

Additionally, for testing the recoverability in bimodal genes, measurements were performed at 4 different stages. Namely, cells were extracted after 100, 150, 300, 500 min in early exponential growth phase, exponential growth phase, early stationary phase, and stationary phase respectively, and flow cytometry measurements are performed. Moreover, cells were inoculated in new medium upon reaching stationary growth phase (first, second and third generations).

- 1. **Microscopy and image analysis**

All 7 strains that were classified as having bimodal distributions of gene expression from flow-cytometry data, were subsequently imaged by microscopy when in standard growth conditions. For comparison, strains containing fluorescently tagged *appC* and *bcsE*, respectively, were also imaged as representatives of genes with unimodal gene expression distribution.

Cells were pelleted and re-suspended in ~100 µL of the remaining media. Then 3 µL of cell suspension were placed on a 2% agarose gel pad made up of M9 Glucose medium and kept in between the microscope slide and a coverslip. It took ~5 min to move cells from the incubator to the microscope and start the observations. This time includes the assembly of the microscope imaging chamber containing the slides and cells. YFP-tagged strains were visualized by confocal microscopy with a 100x objective using a 488 nm laser and a 514/30 emission filter. Simultaneously, phase-contrast images were taken by an external phase-contrast system.

The phase contrast images of the genes *appC,* *bcsE,* *metK*, and *pyrH* were used to detect cell borders using the ‘CellAging’ software [6] based on Gradient Path Labelling [7]. Next, the same software automatically aligned the segmentation results with the corresponding fluorescence images. Overall, we analyzed approximately 500 cells of each YFP-tagged strain.

To investigate the spatial distributions of the fluorescent protein inside the cells, we converted the fluorescence matrix of each cell into a 10 by 10 matrix (using the MATLAB function *imresize*). Next, normalized each cell fluorescent intensity by its maximum value. Finally, we calculated average normalized intensity from all cells of each strain.

- 1. **Classifying distributions as unimodal or bimodal**

We classified a gene as having bimodal gene expression if its single-cell protein distribution complies with one of the 3 classification methods.

First, we selected manually which distributions have a bimodal shape. We minimized classification subjectivity by asking 15 individuals to classify the distributions as bimodal or unimodal (no other information was shared apart from the distributions). The distributions were classified as bimodal if more than 90% of the participants classified them as bimodal.

Second, instead, we fitted a gamma distribution to the positive FITC-H value with the MATLAB function *fitdist*. We also fitted a bimodal distribution (two peaks), with the number of peaks detected using 100 iterations of the MATLAB function *fitgmdist*. This function fits a Gaussian mixture distribution model (with 2 components) and outputs the means (*µ_1_* and *µ_2_*) and standard deviations (*σ_1_* and *σ_2_*) for the 2 Gaussian distributions that best fit the data. To ensure that the detected distributions are far apart enough, we set that condition (S1) holds true [8]:

$\left| \mu_{2}-\mu_{1} \right| \leq S\left( r \right)\cdot\left( \sigma_{1}+\sigma_{2} \right)$ (S1)

In (S1), *S*(*r*) is the separation factor, $S\left( r \right)=\frac{\sqrt{-2+3r+3r^{2}-2r^{3}+2\left( 1-r+r^{2} \right)^{\frac{3}{2}}}}{\sqrt{r}\left( 1+\sqrt{r} \right)}$, and *r* = (*σ_1_* / *σ_2_*)^2^.

We then calculated the BIC for each fit and determined the mean BIC for the biological replicates (BIC_unimodal_ and BIC_bimodal_) and classified the distribution as bimodal if BIC_unimodal_ - BIC_bimodal_ > 10, which usually occurs when one function fits much better than the other [9]. This is expected to happen when the distribution has an uncommon shape (such as ‘shoulder’ or a ‘tail’) which, in turn, would occur when there are two expression modes whose peaks are close to each other.

Finally, third, we instead fitted a non-parametric distribution to the protein expression levels (Section S1.5). Next, we detected local peaks in the distribution by applying the MATLAB function *findpeaks* to the fitting results. This function obtains each peak’s prominence (which measures how much the peak stands out due to its intrinsic height and its location, relative to other peaks). We classified peaks as true positives only when the ratio between its prominence and the maximum prominence in the distribution was higher than 1%, as it sufficed to remove spurious peaks (Fig V in S1 Text). Finally, we classified genes as having bimodal dynamics if two true peaks were found in at least two biological replicates.

For the genes classified as bimodal, we extracted the 3 bimodal distributions from each of the classification methods. We calculated the value for *d*, *h*, and *o* (Eqs. 1-3 in the main manuscript) as the median value calculated from the 3 distributions.

- 1. **Fittings and statistical analysis**

To find correlations between two variables, we performed regression models. The correlation was considered significant if the estimated coefficients are significantly different from 0 (i.e., for *p*-values < 0.05 of the *t*-test).

Meanwhile, to test if the means of 2 distributions are significantly different, we used two-sample *t*-tests, whose null hypothesis is that the distributions have equal means. For *p*-values < 0.05, we considered that the distributions have significantly different means.

Finally, nonparametric fittings to single-cell distributions were done using MATLAB´s function *fitdist* with a normal kernel smoothing function. Meanwhile, the joyplots in the figures were created using R’s ‘ggridges’ package.

- 1. **Simulations**

Modelling and simulations were performed using the simulator ‘SGNS2’ [10], whose dynamics follows the Stochastic Simulation Algorithm [11]. Reactions occur within hierarchical, interlinked compartments which can be created, destroyed, and divided at runtime.

The time length of each simulation was set to 5×10^5^ s as it sufficed to reach quasi-equilibrium. Data on the system state was collected each 100 s. Also, 100 independent runs were performed for each set of parameter values (Table B in S1 Text), as it sufficed to overcome potential variabilities due to small numbers of simulations. In each simulation, the initial state of the promoter was set randomly, with equal probability of being in the “L” or “H” state. Nevertheless, we also discarded the first 1000 data points (20% simulation time), so that the protein number distributions were obtained from time points when the systems already reached quasi-equilibrium in either state. The rate constant values controlling the state transitions in the control condition allowed for an average of 2.5 state transitions in each simulation.

- 1. **Estimation of rate constants reference values**
     1. **Decay rates**

In [12], it is reported that, in standard growth conditions exponentially growing *E. coli* cells exhibit mean average RNA lifetimes of 4.1 min. From this, we estimate average RNA degradation rates ($\gamma$_RNA_) of:

$\gamma_{RNA} = \frac{1}{4.1\cdot60}=0.0041 s^{-1}$ (S2)

Meanwhile, [13] reported that the bulk of the steady-state protein pool degrades at the rate of ~1%/hour [13]. Thus, we assume average protein degradation rates ($\gamma$_P_) to be:

$\gamma_{P} = -\frac{ln(0.99)}{3600}=2.79\times{10}^{-6} s^{-1}$ (S3)

Next, we considered that both RNA and proteins are diluted by cell division. To estimate the dilution rates, we estimated cell doubling times, *D*, from optical density values over time (Fig U in S1 Text). From that, we estimated the dilution rate, $\gamma_{dil}$, of both RNA and proteins to be:

$\gamma_{dil} =\frac{\ln\left( 2 \right)}{D}=5\times{10}^{-3}{min}^{-1}$ (S4)

Finally, we estimate the consequent RNA and protein decay rates (due to degradation and dilution by cell division, *k*_d_^RNA^ and *k*_d_^P^ respectively) to be:

${k_{d}^{RNA}=\gamma_{RNA}+\gamma}_{dil}= 0.004s^{-1}$ (S5)

${k_{d}^{P}=\gamma_{P}+\gamma}_{dil}= 8.65\times{10}^{-5}s^{-1}$ (S6)

- - 1. **Transcription rates**

From [13] the mean RNA numbers per gene, *M_RNA_*, under standard growth conditions is 0.4 per cell. Thus, considering the value of *k_d_^RNA^* (Eq. S5) and assuming 144 RNAPs freely available at any given time [14], from Eq. S7, we estimate the average RNA production rate *k_t_* to equal 1.15×10^-5^ s^-1^.

$k_{t}\times RNAP=M_{RNA}\cdot k_{d}^{RNA}$ (S7)

In this regard, σ^70^ numbers and the rate constants controlling holoenzymes are set so as not to be rate limiting factors of RNA production, by ensuring that nearly all free floating RNAPs are always in holoenzyme form (Table B in S1 Text) [15–17]. For example, σ^70^ numbers are set to double the amount of core RNAPs.

Next, assuming the reference value extracted from Eq. S7, we set a “low” transcription rate, *k_t_^L^*, to be 1.5× lower (7.68×10^-6^ s^-1^). This value sufficed to obtain a gaussian-like shape for the distribution of protein numbers at the low expression state, in agreement with our observations (Fig 2 in the manuscript). Lower values would result instead in an exponential-like distribution, which we only observed in 1 of our 42 measurements (Fig 2B in the manuscript, stationary growth condition).

Similarly, we set a “high” transcription rate, *k*_t_^H^, to be 5× larger (5.76×10^-5^ s^-1^) than the reference value because many genes are capable of maintaining ≈1000 proteins in the cell at any given moment, while higher values than that are rare [17].

To impose these rates in the model with 2 rate limiting steps in transcription (Fig 6A in the manuscript), we consider that:

$\frac{1}{k_{t}\times RNAP}=\frac{1}{k_{bind}\times RNAP}+\frac{1}{k_{esc}}$ (S8)

Further, for simplicity, in both transcription states, we assume that the total time between expected RNA production events is divided equally between promoter binding and promoter escape, as follows:

$\frac{1}{k_{bind}^{H}\times RNAP}=\frac{1}{k_{esc}^{H}}=\frac{1}{k_{t}^{H}\times RNAP}\cdot\frac{1}{2}$ (S9)

$\frac{1}{k_{bind}^{L}\times RNAP}=\frac{1}{k_{esc}^{L}}=\frac{1}{k_{t}^{L}\times RNAP}\cdot\frac{1}{2}$ (S10)

- - 1. **Rates of σ factor preference**

To model the stationary growth phase, the model allows increasing the concentration of σ^38^. By having two competing reactions to form RNAP.σ^70^ and RNAP.σ^38^, along with the reversible reactions, the ratio of these two holoenzymes numbers will equal the ratio between σ^70^ and σ^38^ numbers. Consequently, the mean values of the holoenzymes RNAP.σ^38^ (M_RNAP.σ38_) and RNAP.σ^70^ (M_RNAP.σ70_) can be estimated from:

$M_{RNAP.\sigma^{70}}=\mathrm{RNAP}\cdot\frac{\sigma^{70}\cdot k_{70}}{\sigma^{70}\cdot k_{70}+\sigma^{38}\cdot k_{38}}$ (S11)

$M_{RNAP.\sigma^{38}}=\mathrm{RNAP}\cdot\frac{\sigma^{38}\cdot k_{38}}{\sigma^{70}\cdot k_{70}+\sigma^{38}\cdot k_{38}}$ (S12)

During standard growth conditions, the only σ factor that is present in substantial amounts is the housekeeping σ factor, σ^70^ [18]. Thus, the [RNAP.σ^70^]/[RNAP] ratio will equal 1, as the number of σ^70^ factors is nearly double the amount of core RNAPs [19].

However, during the stationary growth phase, there is also σ^38^ factors in the cells. Namely, the ratio [σ^38^]/[σ^70^] is ≈22% [19], while the ratio [RNAP.σ^38^]/[RNAP.σ^70^] is ≈24% [20]. Given this, we set the ratio between the rate constants controlling holoenzymes formation to:

$\frac{k_{38}}{k_{70}}=\frac{\left( \frac{M_{RNAP.\sigma^{38}}}{M_{RNAP.\sigma^{70}}} \right)}{\left( \frac{\sigma^{38}}{\sigma^{70}} \right)}=\frac{0.24}{0.22}=1.1$ (S13)

The value of each rate is shown in Table B in S1 Text.

- - 1. **Translation rate**

Given the model, the steady state solution for mean protein numbers (*M*_P_), is given by:

$k_{tr}=\frac{M_{P}}{M_{RNA}}\cdot k_{d}^{P}$ (S14)

Since, on average, 540 proteins are produced per RNA [21], while average protein decay rates equal 8.66×10^-5^ s^-1^ (Eq. S6), we have set *k_tr_* to 0.047 s^-1^.

- - 1. **Rates of state transitions**

The rates *k_L_* and *k_H_*, control, respectively, the expected time for a promoter with high expression rate to transit into low expression rate, and vice versa. While empirical data for these rate constants is lacking, the model suggests that the existence of bimodality implies that they are slow, relative to other rate constants of the model. For example, the rate needs to be slower than the time for the proteins of either gene to almost entirely degrade if one is to have time intervals differing significantly in mean protein numbers. Here, we assumed protein decay rates of approximately 10^−5^ s. Thus, if the system contains ~500 proteins in a given state [21], it would take 10^5^ s for most of them to degrade, following the gene’s repression. Consequently, we set the switching time to 10^5^ seconds by assigning *k_L_* and *k_H_* ​ values of 0.00001 s^-1^.

Also, these state transitions are independent of any other events in the model, except for the amount of time that promoters stay bound to RNAPs. Namely, during those time periods, promoters cannot switch states. Thus, differences in the duration of those periods differs between high and low expression states introduce weak asymmetries in the probabilities for state transitions. For example, if *k_esc_* is higher for the high expression state, while *k_L_* and *k_H_* are identical, it will be slightly more likely to find the promoter in the weak expression state than in the high expression state. This difference can be compensated for by making *k_H_* slightly higher than *k_L_*.

**Supplementary Figures**


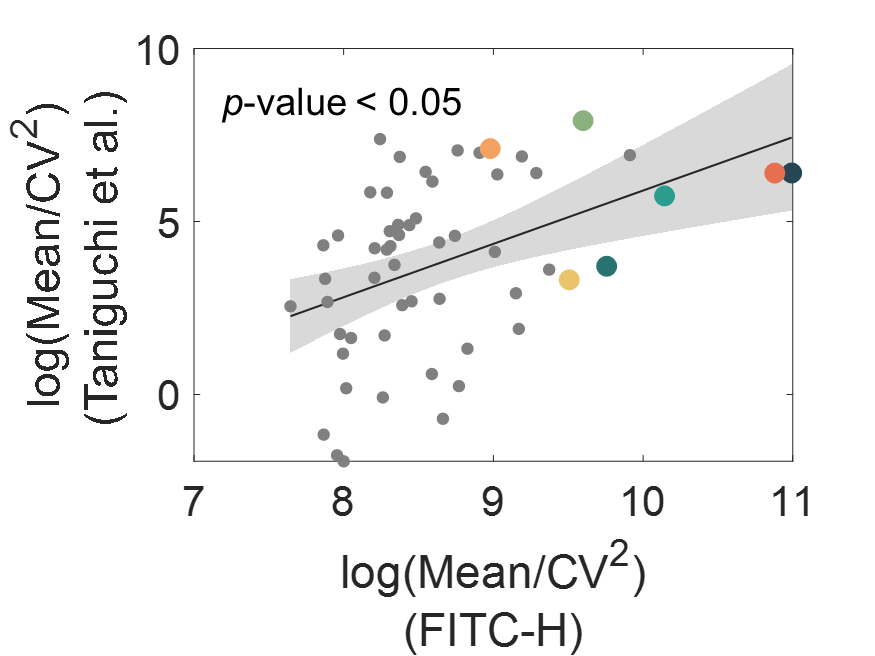


**Fig A:** Scatter plot of the ratio between mean and noise (CV^2^) from flow cytometry data plotted against the same ratio reported in [1]. Also shown is a best fitting line (and the corresponding *p*-value). Shadow areas are 95% confidence bounds. Data points corresponding to distributions considered bimodal are colored for easier visualization.

**Fig B:** Single-cell distributions of fluorescent levels (FITC-H) from Fig 1 in the main manuscript. Each distribution results from merging three biological repeats.


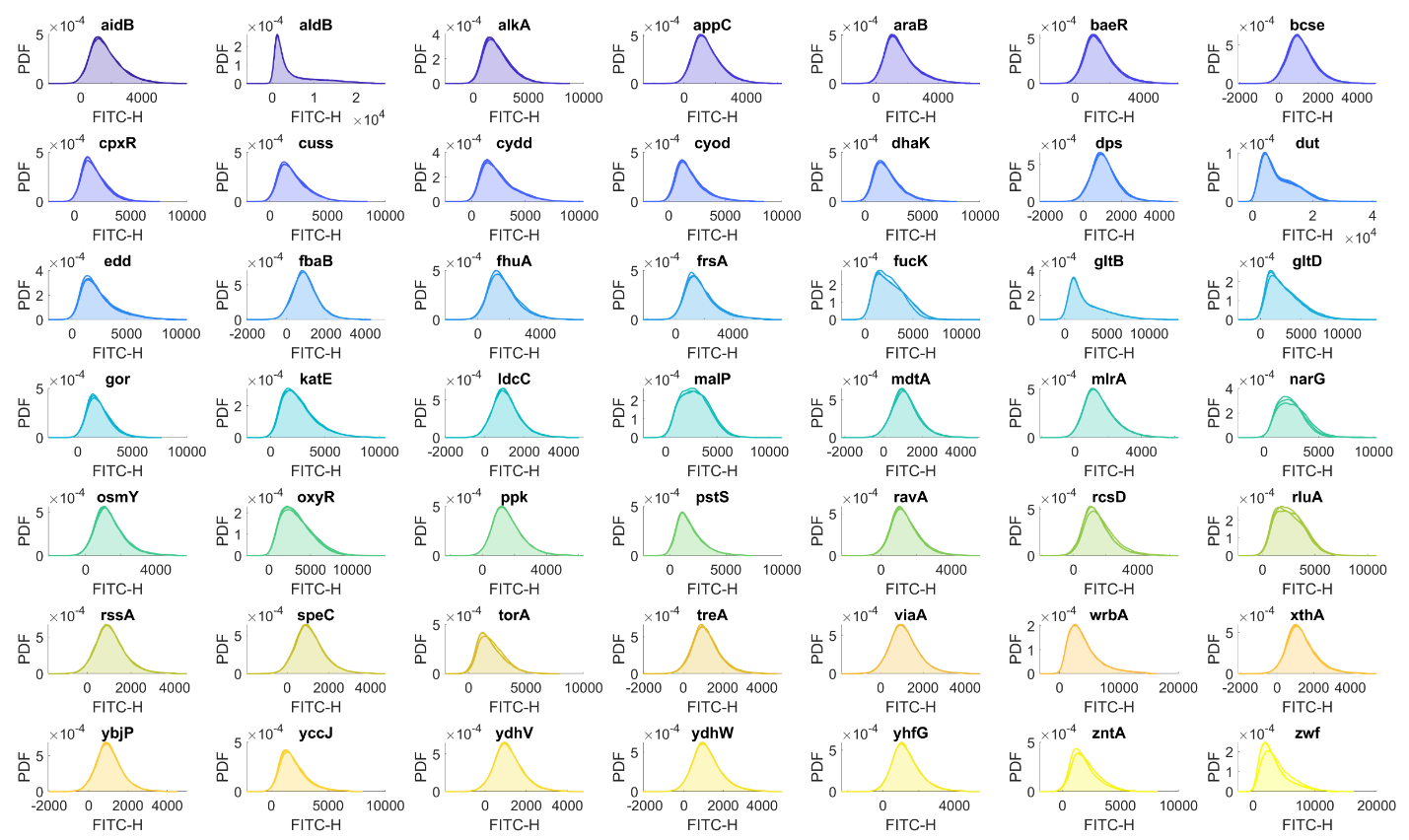


**Fig C:** Probability density functions (PDF) of the single-cell distributions of protein expression levels (FITC-H) classified as unimodal. Each plot contains 3 distributions corresponding to 3 biological replicates, respectively. Also shown are the names of the genes.

**Fig D:** Probability density functions (PDF) of the single-cell distributions of protein expression levels (FITC-H) classified as bimodal. Each plot contains 3 distributions corresponding to 3 biological replicates, respectively. Also shown are the names of the genes.


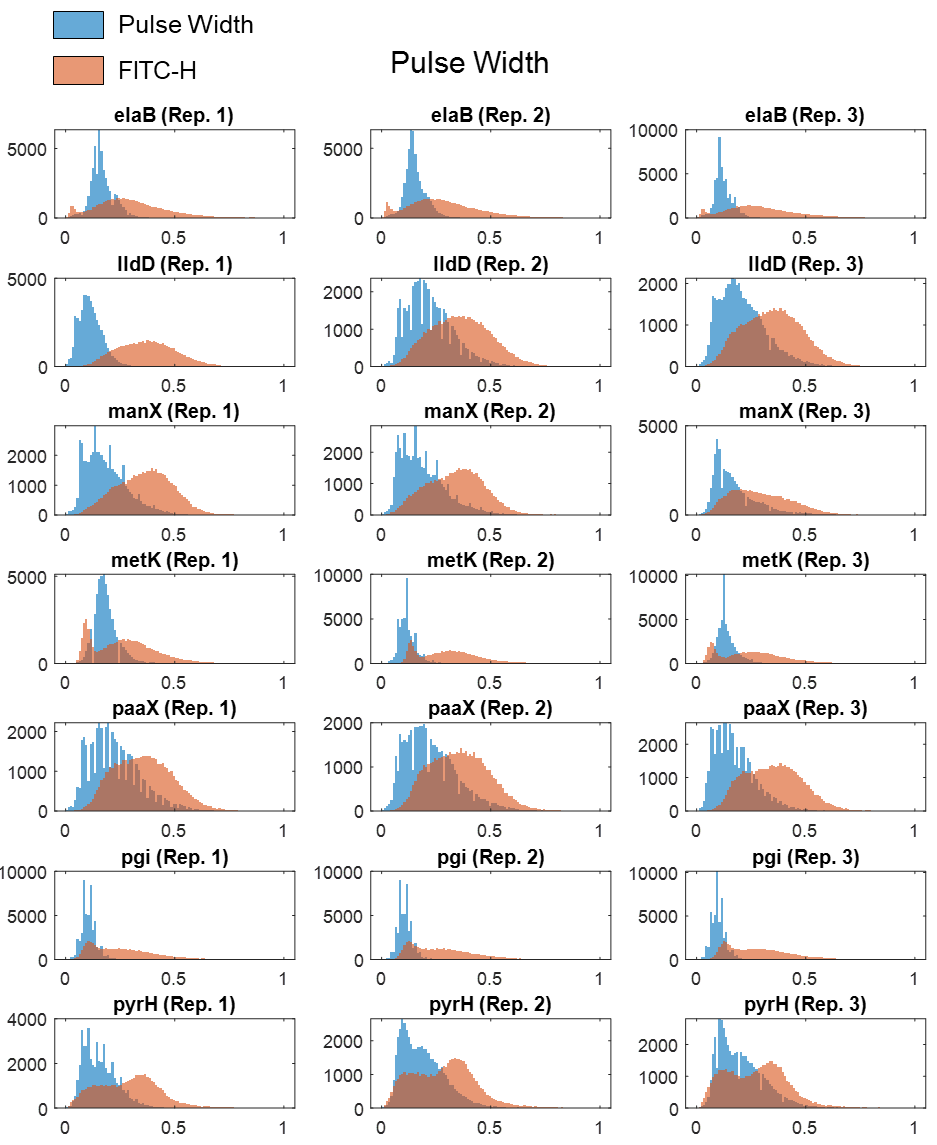


**Fig E:** Normalized single-cell distributions of protein expression levels (orange) and of pulse width (blue), respectively, as measured by flow-cytometry. Each distribution is from 1 biological replicate (‘Rep.’). For visualization purposes, “far-out” events (as classified by Tukey fences) were discarded from the Pulse Width distributions. Also shown are the names of the genes.


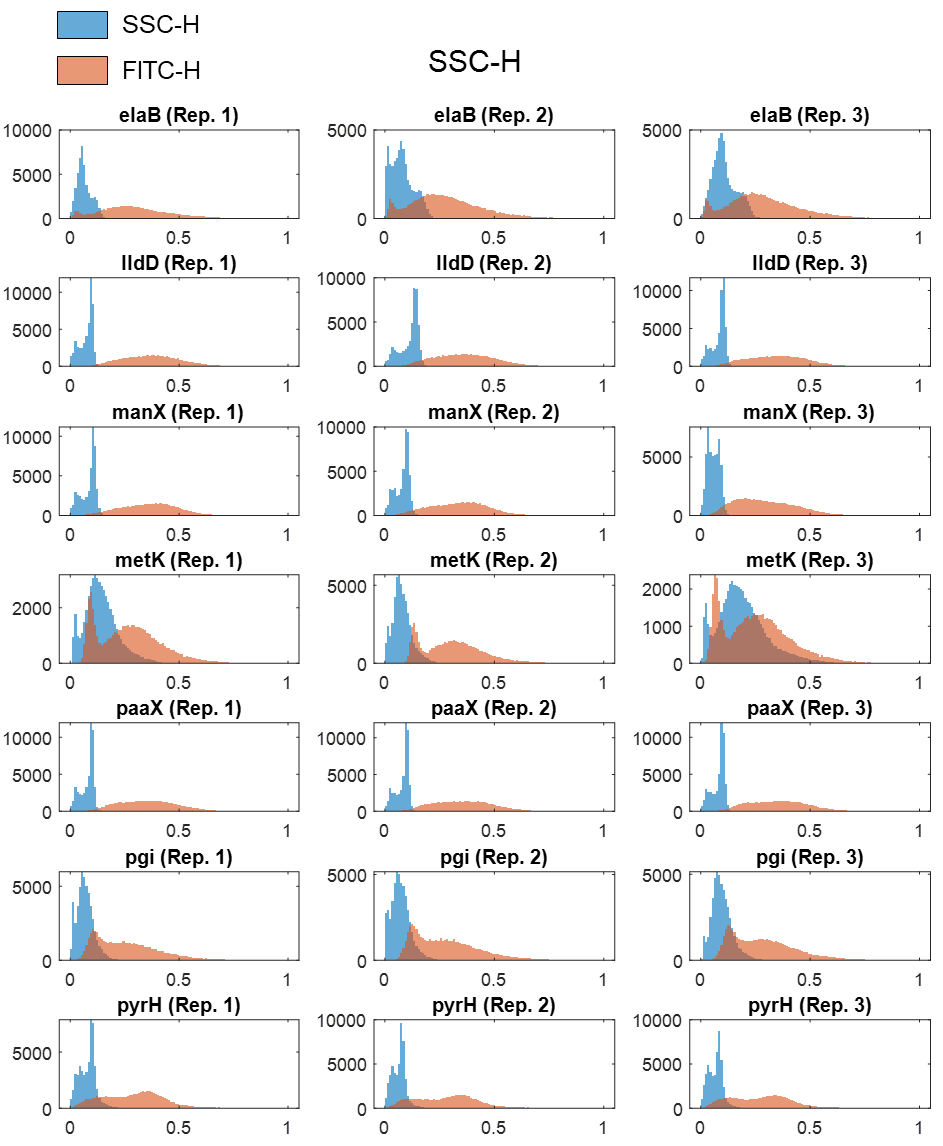


**Fig F:** Normalized single-cell distributions of protein expression levels (orange) and of FSC-H (blue), respectively, as measured by flow-cytometry. Each distribution is from 1 biological replicate (‘Rep.’). For visualization purposes, “far-out” events (as classified by Tukey fences) were discarded from the SSC-H distributions. Also shown are the names of the genes.


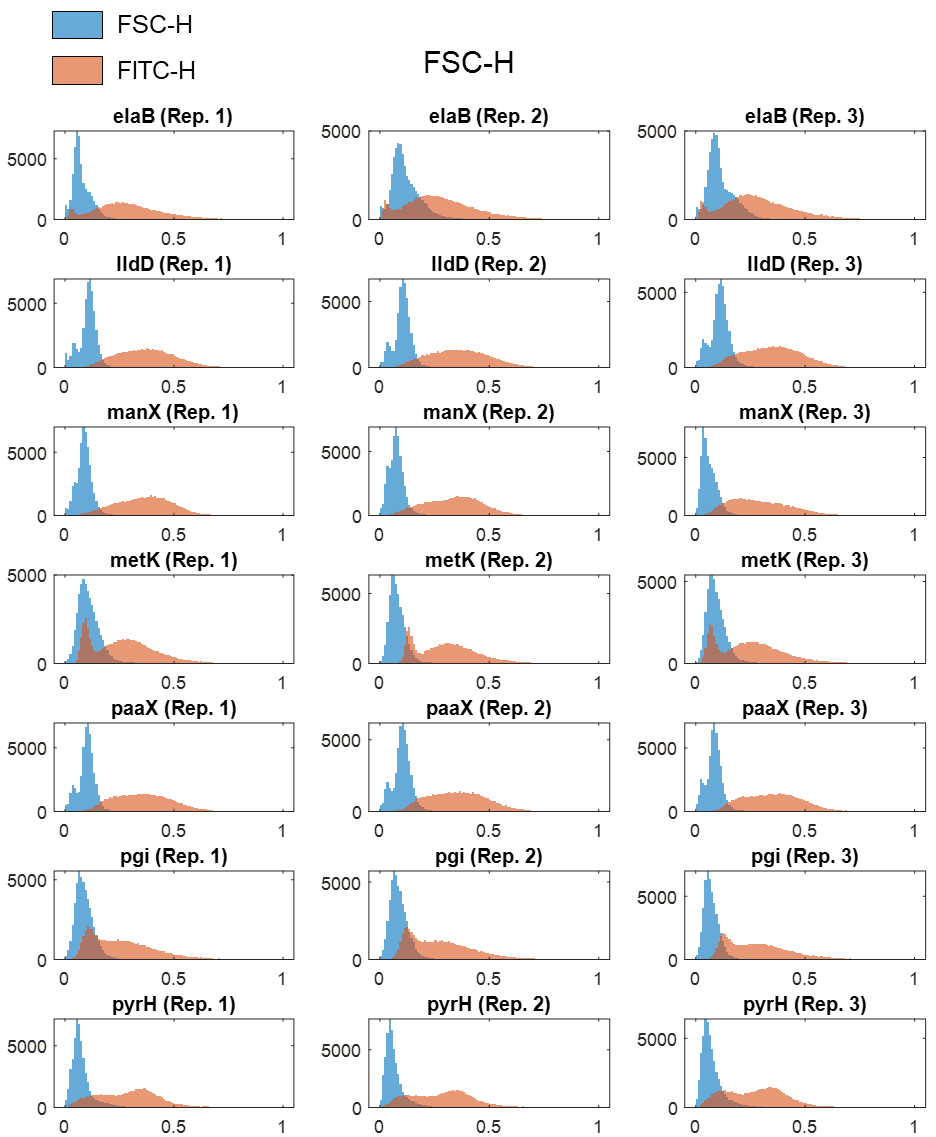


**Fig G:** Normalized single-cell distributions of protein expression levels (orange) and of SSC-H (blue), respectively. Each distribution is from 1 biological replicate (‘Rep.’). For visualization purposes, “far-out” events (as classified by Tukey fences) were discarded in the FSC-H distributions. Also shown are the names of the genes.


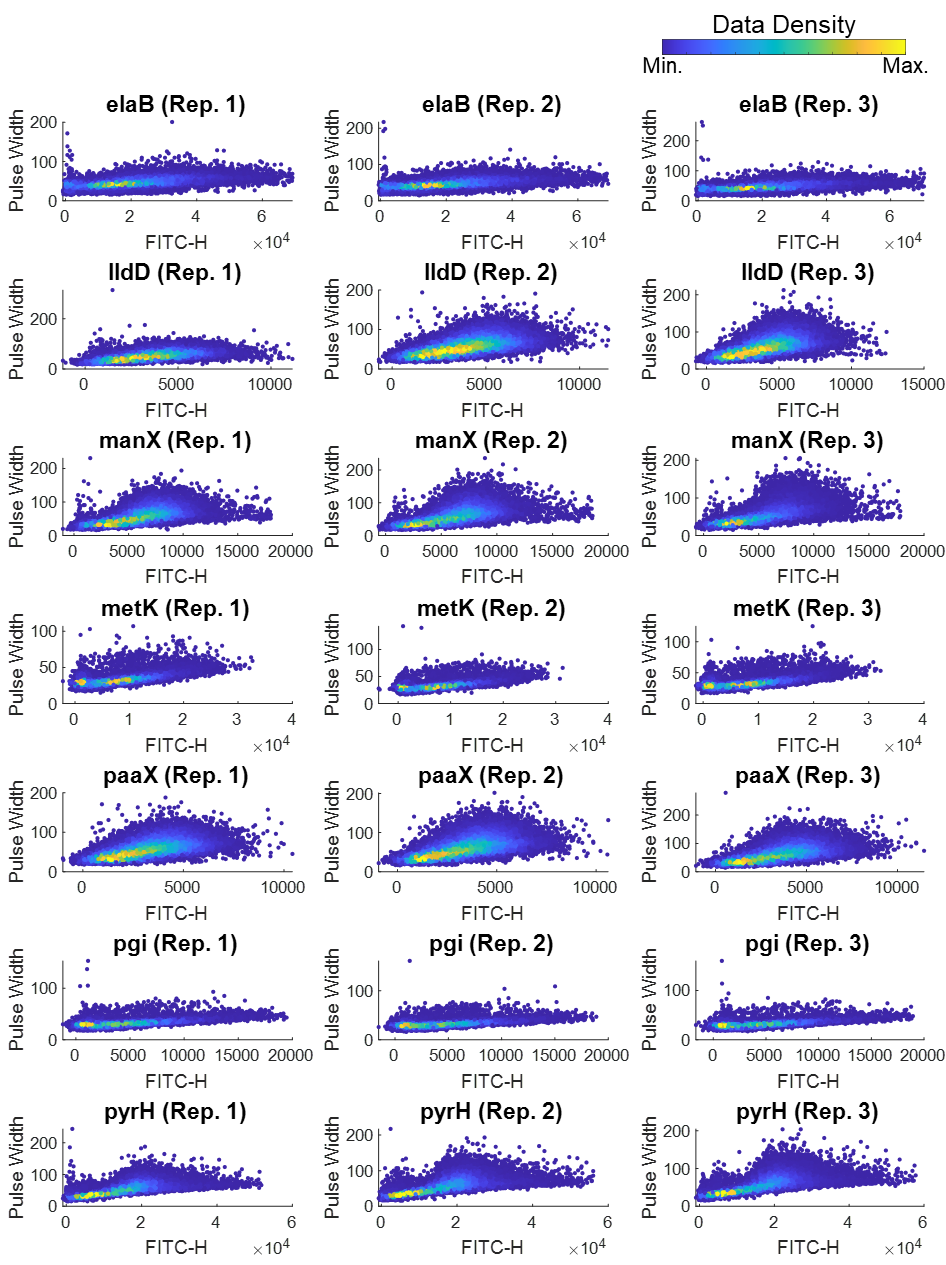


**Fig H:** Scatter plot between single-cell protein expression levels (FITC-H) and of pulse width, as measured by flow-cytometry. The color of the data points indicates data density. Each distribution is from 1 biological replicate (‘Rep.’). For visualization purposes, “far-out” events (as classified by Tukey fences) were discarded from the Pulse Width distributions. Also shown are the names of the genes.


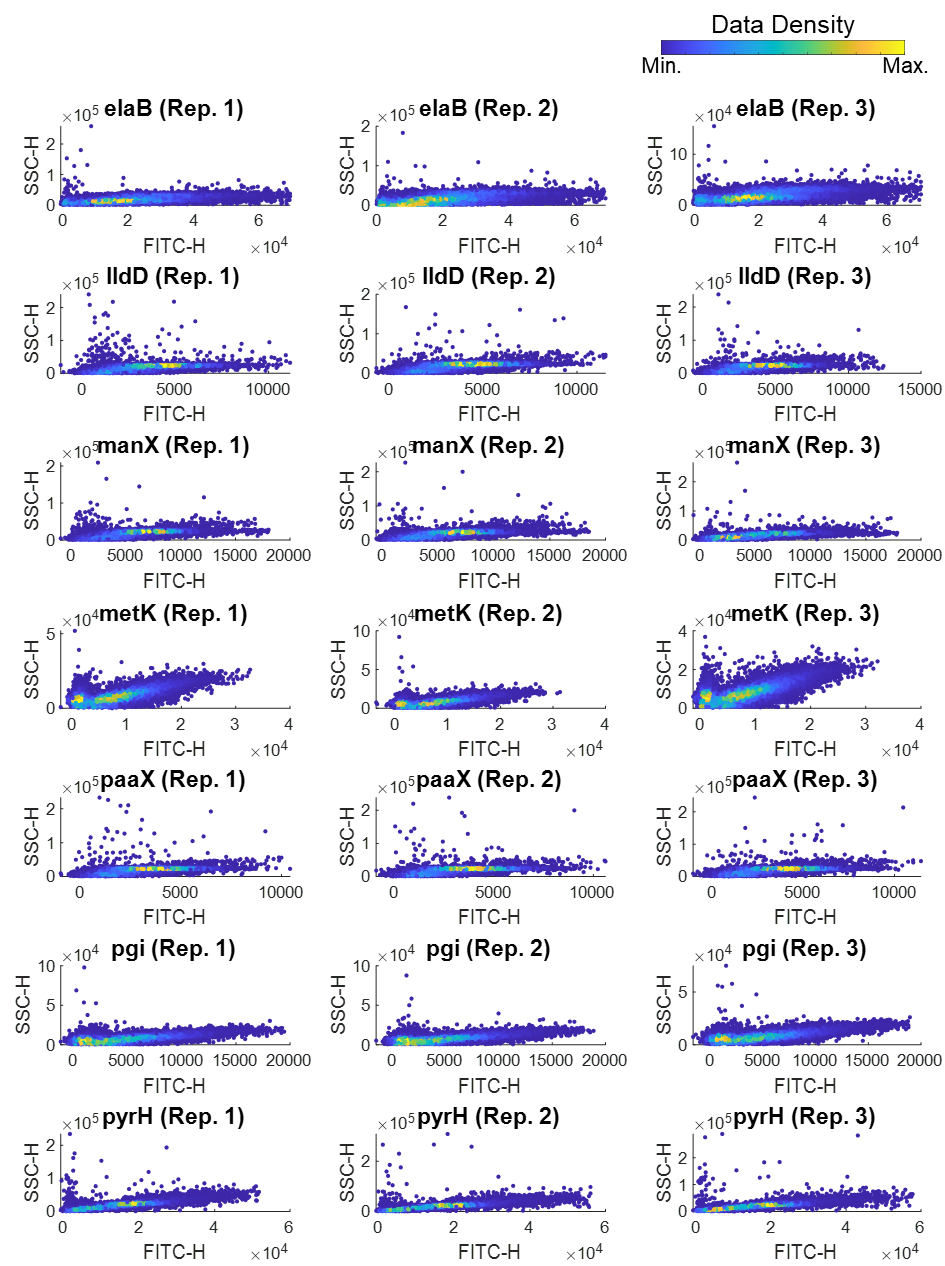


**Fig I:** Scatter plot between single-cell protein expression levels (FITC-H) and of SSC-H, as measured by flow-cytometry. The color of the data points indicates data density. Each distribution is from 1 biological replicate (‘Rep.’). For visualization purposes, “far-out” events (as classified by Tukey fences) were discarded from the Pulse Width distributions. Also shown are the names of the genes.


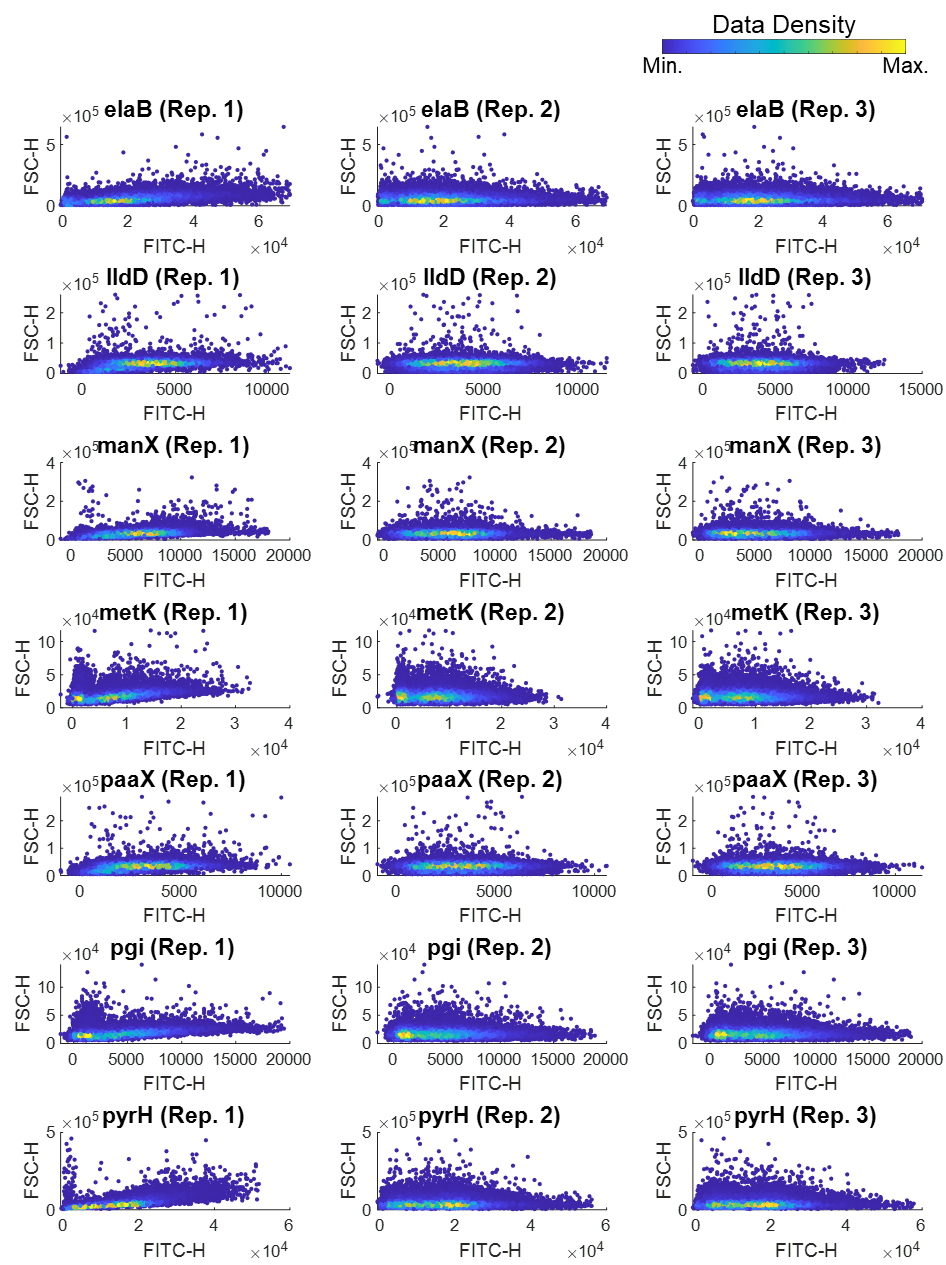


**Fig J:** Scatter plot between single-cell protein expression levels (FITC-H) and of FSC-H, as measured by flow-cytometry. The color of the data points indicates data density. Each distribution is from 1 biological replicate (‘Rep.’). For visualization purposes, “far-out” events (as classified by Tukey fences) were discarded from the Pulse Width distributions. Also shown are the names of the genes.

**
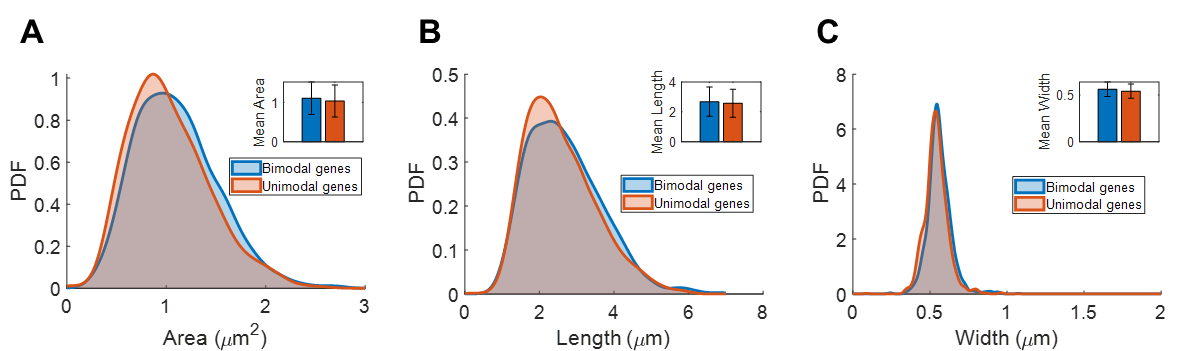
**

**Fig K:** Probability density functions of sizes of cells with fluorescently tagged genes with bimodal protein distributions (*metK* and *pyrH*) and with unimodal protein distributions (*appC* and *bcsE*). **(A)** cell areas, **(B)** cell lengths and, **(C)** cell widths. In all plots, the insets show the mean value of the distributions. The error bars represent the standard deviation. We analyzed approximately 500 cells of each YFP strain.


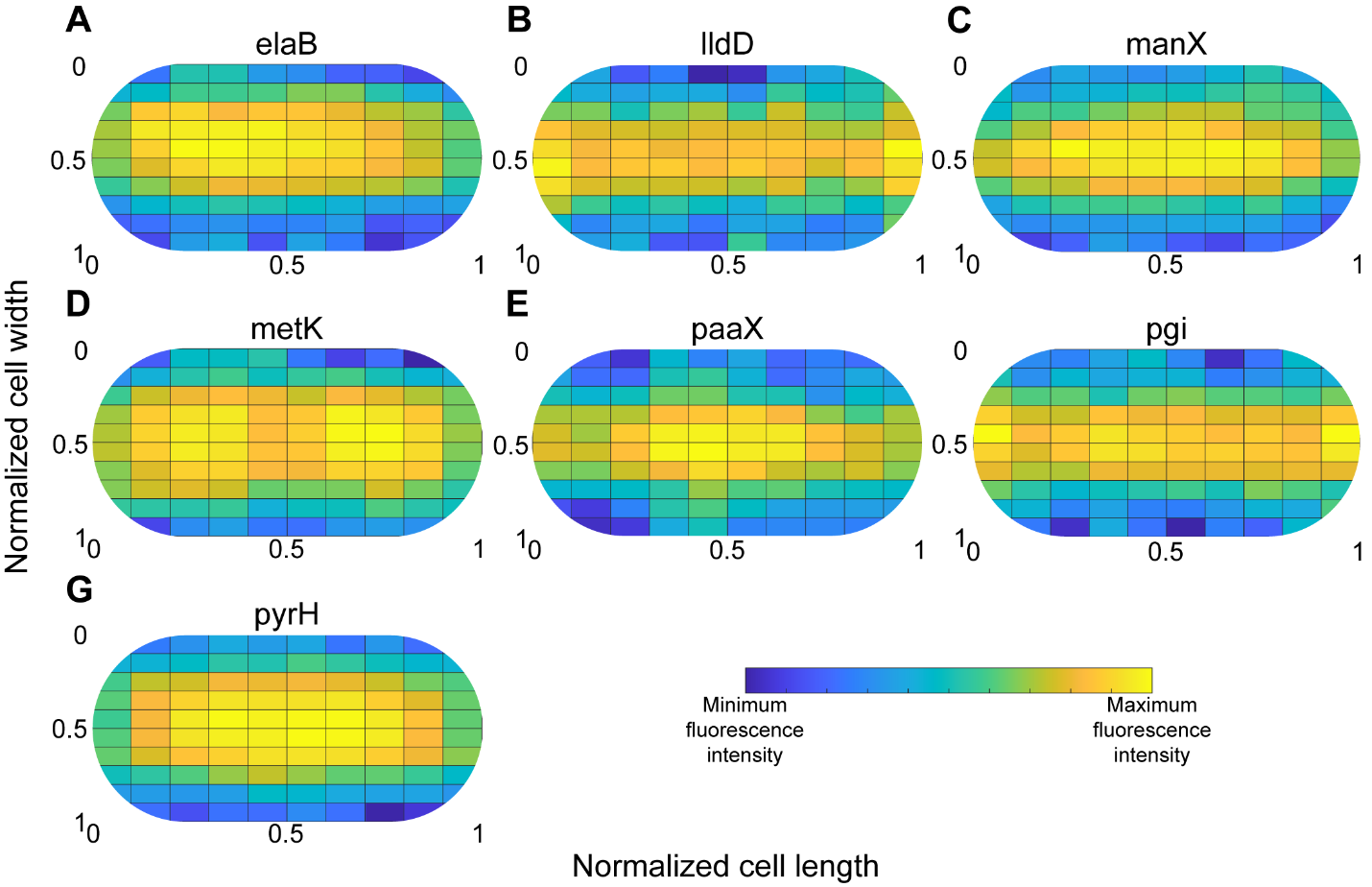


**Fig L:** Cellular spatial distributions of fluorescence intensity of proteins with single-cell bimodal distributions of expression levels. Shown are the mean fluorescence intensities (normalized by the maximum intensities) of the various regions of the cells. Cell sizes were normalized. First, microscopy images were collected. Next, cell areas were normalized and then split into 10x10 sub-areas. Then, mean intensities of each area were obtained. Then, they were normalized by the highest of the intensities. Genes names are shown on top of each image.


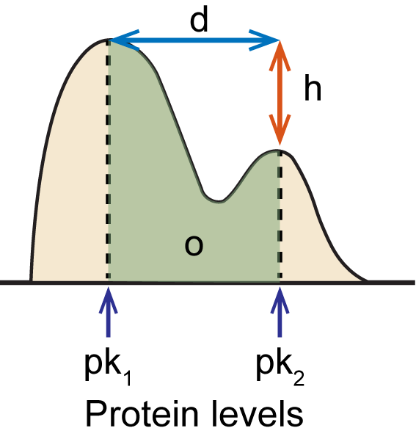


**Fig M:** Illustration of the parameters used to characterize bimodal distributions: *d*, *h*, and *o*.

**
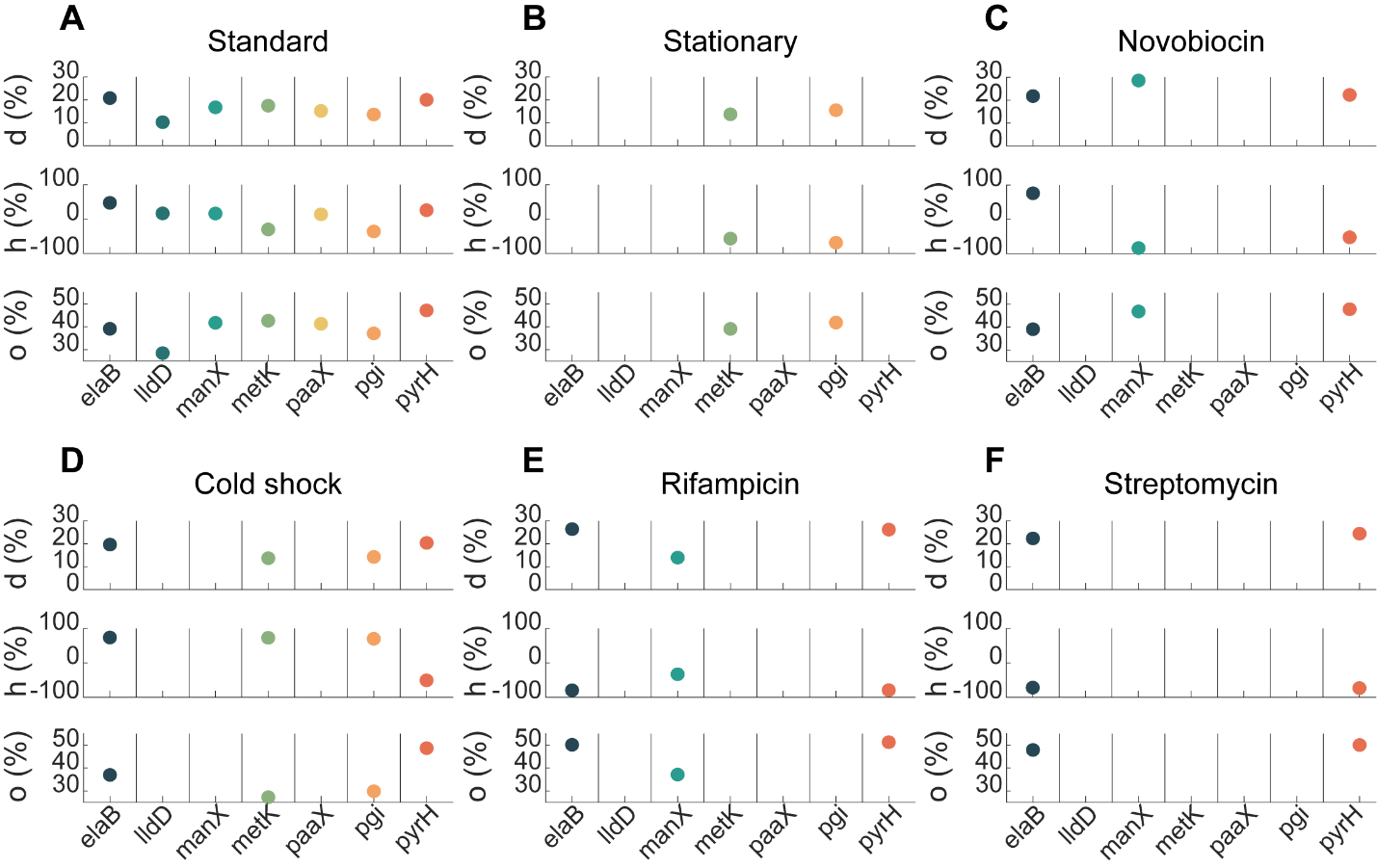
Fig N:** Parameter values of the bimodal distributions of single-cell protein levels in cells in: **(A)** standard growth conditions, **(B)** stationary growth phase, **(C)** under novobiocin, **(D)** under cold shock, **(E)** under rifampicin, and **(F)** under streptomycin. Values are not plotted if the distributions were classified as unimodal.


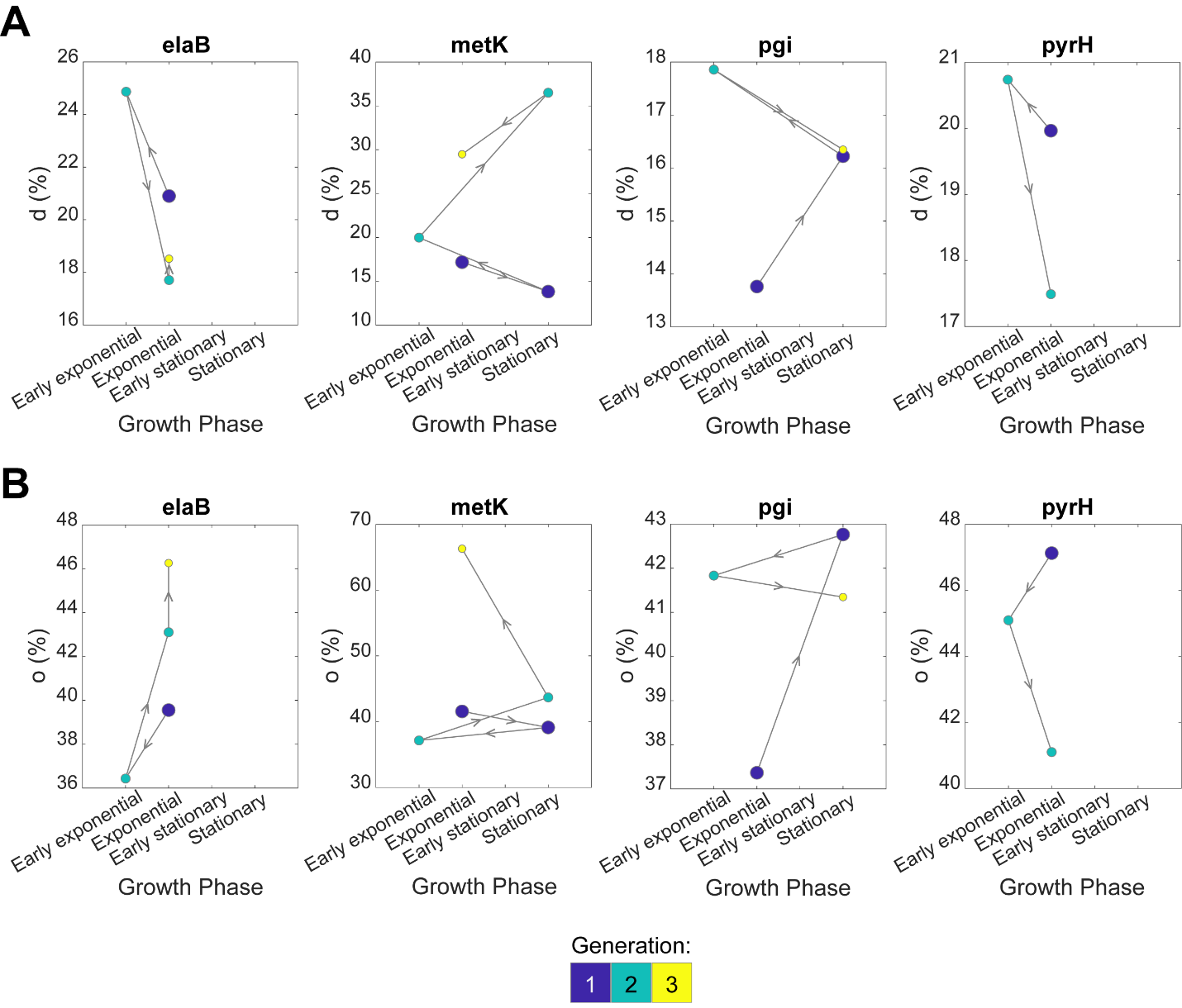


**Fig O:** Evolution of the values of **(A)** *d* and **(B)** *o*, used to characterize the shape of the bimodal distributions of single-cell protein levels across growth phases (early exponential, exponential, early stationary and stationary) and generations. Only genes with bimodal distributions in more than one growth phase were considered and the values were not plotted if the distributions were classified as unimodal in a given phase. The arrows in the lines connecting the data points represent the progression of time. The dark blue, teal and yellow data points represent the 1^st^, 2^nd^ and 3^rd^ generation, respectively.


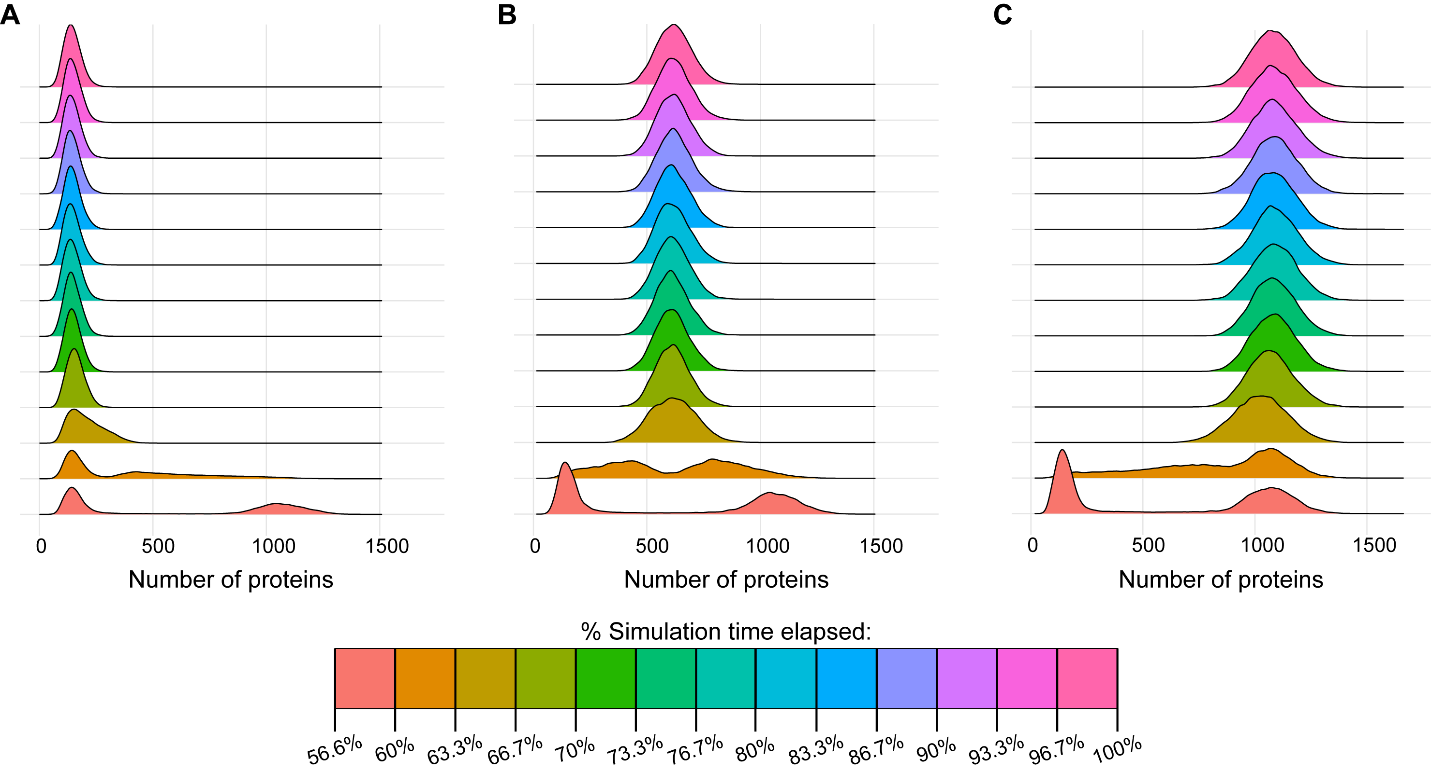


**Fig P:** *In silico* single-cell distributions of protein numbers estimated from simulations. Three models are considered, with the expression rate of the only state being set to equal **(A)** the rate of original state with lower-expression rate; **(B)** the average of the rates of the two original states; and **(C)** the rate of the original state with high-expression. In all cases, we start simulations with a system with two states, which collapses into one at 60% of the simulation time. Visibly, it still takes some time after that for the bistability to no longer be detectable.


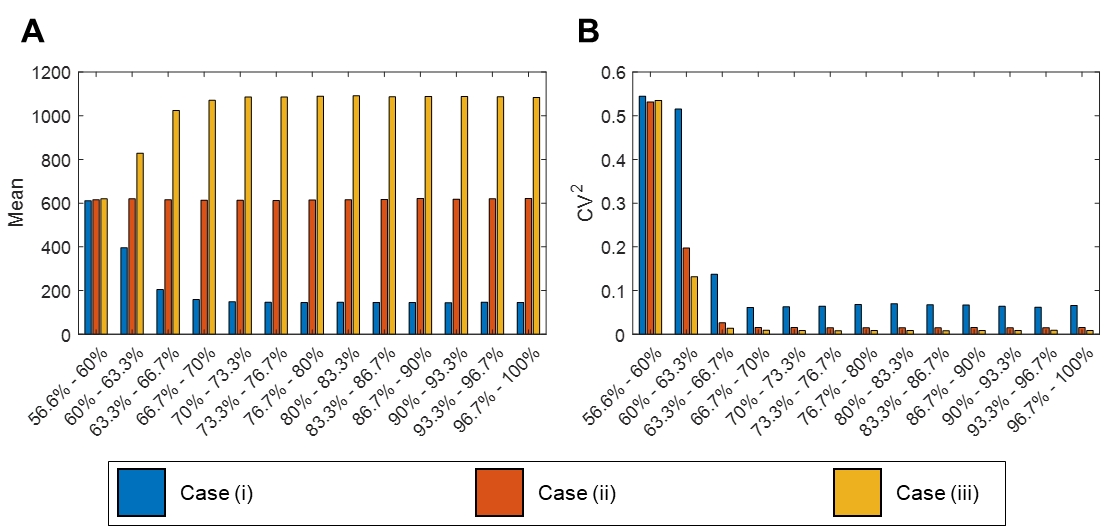


**Fig Q:** Evolution of **(A)** mean and **(B)** noise (CV^2^) of *in silico* single-cell distributions of protein numbers estimated from simulations of the models of bistability. In all cases, we start simulations with a system with two states, which collapses into one at 60% of the simulation time, with expression rate of the remaining state being that of: (i) the original low-expression rate (blue bars), (ii) a rate equal to the average of the rates of the two original states (orange bars), and (iii) the original high-expression rate (yellow bars).

**
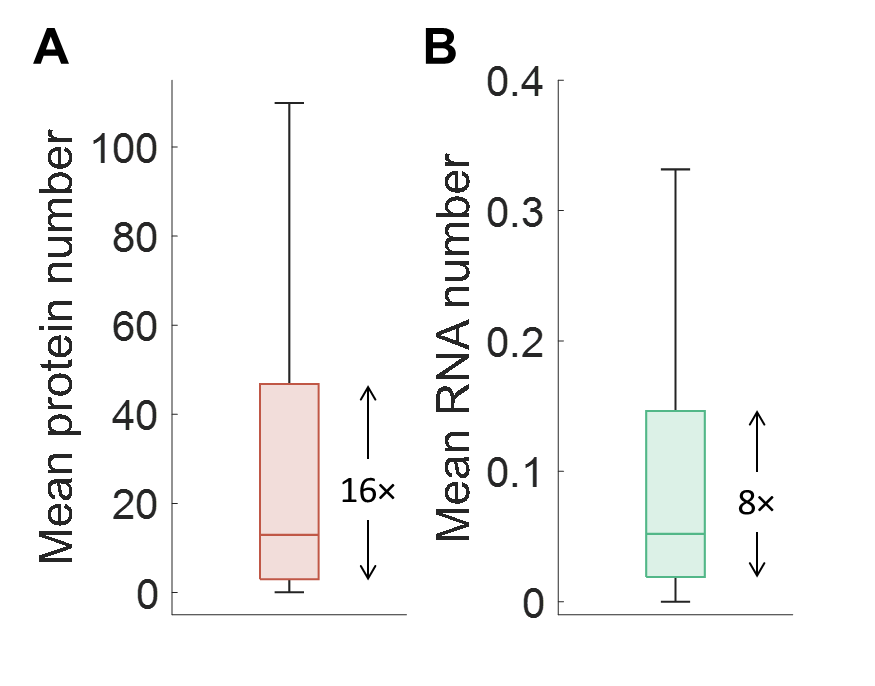
**

**Fig R: (A)** Boxplot of the mean single-cell protein numbers reported in [21]. **(B)** Boxplot of the mean RNA numbers reported in [22]. In both boxplots, the outliers (values that are 1.5× above the upper bound of the IQR or 1.5× below the lower bound of the IQR, where IQR is the interquartile range) are not shown. Also shown are the ratios (16× and 8×) between the lower and upper bounds of the IQR.


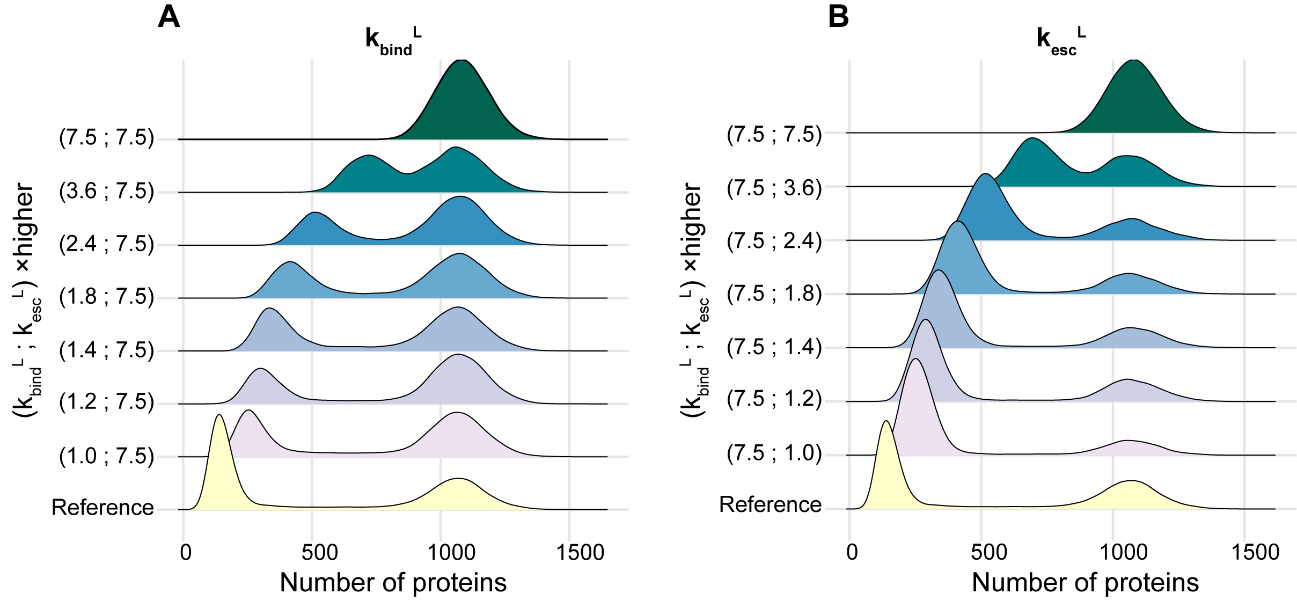


**Fig S:** Single cell distributions of protein numbers estimated from simulations of the reduced model when tuning: **(A)** *k_bind_^L^*, and **(B)** *k_esc_^L^*. Starting from the reference condition (bottom graphs), one parameter was kept constant at 7.5x the reference value, while the other parameter was increased progressively (1.2x, 1.4x, 1.8x, 2.3x, 3.6x, 7.5x).


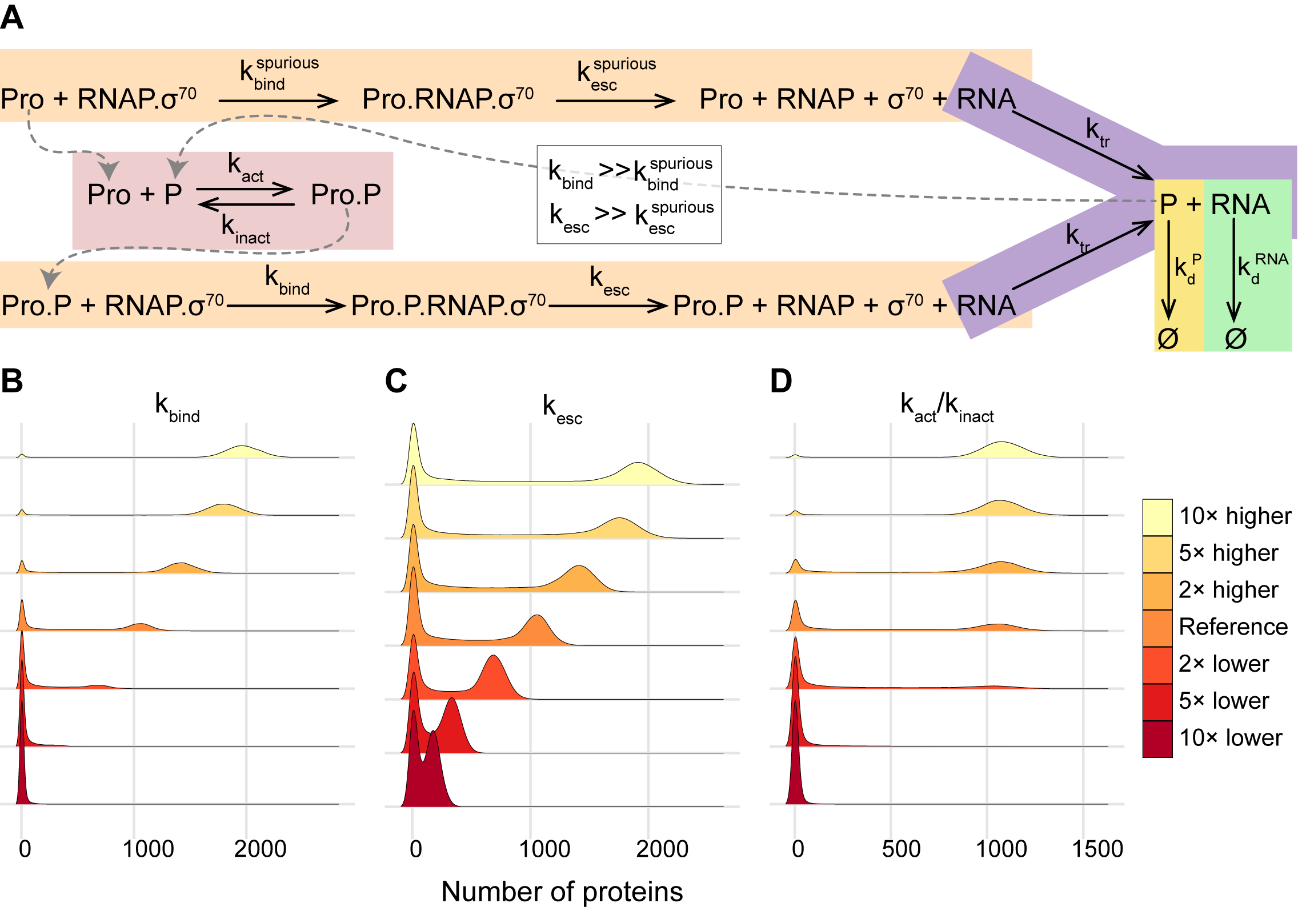


**Fig T: (A)** Model of gene with positive feedback loop regulation. The gene is spuriously (slowly) transcribed (with rates *k_bind_^spurious^* and *k_esc_^spurious^*) so that the system is not “frozen” by an absence of proteins. RNAs are translated into proteins, which can bind and unbind to the gene’s promoter region (at the rates *k_act_* and *k_inact_*, respectively). When bound by a protein, the gene is transcribed with a high frequency (controlled by the rates *k_bind_* and *k_esc_*). Meanwhile, if not bound by a protein, the gene can still be spuriously transcribed (slowly). Finally, there are reactions for RNA and protein degradation. **(B-D)** Single cell distributions of protein numbers obtained from simulations of the positive feedback loop model, at several parameter values for: **(A)** *k_bind_*, **(B)** *k_esc_* **(C)** the ratio *k_act_*/*k_inact_***.** The reference parameter values are shown in in Table D in S1 Text.

**Fig U:** Optical density (O.D._600_) curve of *E. coli* MG1655 cells in M9 medium at 37 °C.

**Fig V:** Number of genes classified as bimodal as a function of the threshold assumed to distinguish between true and spurious peaks. The threshold percentage expresses the minimum prominence value a detected peak must have (relative to the peak with maximum prominence) to be considered a true peak. The orange circle marks the inflection point, as detected by [22], where the curve is best approximated by a pair of lines.

**Supplementary Tables**

**Table A:** Peak values of distributions in Fig 1 in the manuscript. For the bimodal genes, the values of the low and high peaks are the median of the peaks by the methods that classified the distributions as bimodal (manual classification, BIC and peak detection). For WT, the peak value is the median of the peaks detected by the three methods.

| **Name** | **Low FITC-H peak** | **High FITC-H peak** |
| --- | --- | --- |
| WT | 901 | |
| *elaB* | 1768 | 16821 |
| *lldD* | 1919 | 4133 |
| *manX* | 2641 | 6280 |
| *metK* | 1062 | 7309 |
| *paaX* | 1545 | 3271 |
| *pgi* | 1091 | 3996 |
| *pyrH* | 6696 | 18172 |

**Table B:** Parameter values for a reference bimodal model. We ran simulations for rates constants 10, 5, and 2× lower as well as for 2, 5, and 10× higher. The values were selected to be within realistic ranges of parameters values, while also allowing for easy visualization of the influence of each parameter on the distribution.

| **Parameter** | **Parameter Description** | **Value** | **Reference Section** |
| --- | --- | --- | --- |
| **k_L_** | Rate of shifting to state L | 0.00001 s^-1^ | Section 1.7.5 |
| **k_H_** | Rate of shifting to state H | 0.00001 s^-1^ |  |
| **k_bind_^L^** | Rate of promoter binding rate at the state L | 1.54×10^-5^ s^-1^ | Section 1.7.2 |
| **k_esc_^L^** | Rate of promoter escape rate at the state L | 2.21×10^-3^ s^-1^ |  |
| **k_bind_^H^** | Rate of promoter binding rate at the state H | 1.15×10^-4^ s^-1^ |  |
| **k_esc_^H^** | Rate of promoter escape rate at the state H | 1.66×10^-2^ s^-1^ |  |
| **k_tr_** | Translation rate | 0.047 s^-1^ | Section 1.7.4 |
| **k_d_^RNA^** | RNA decay rate (degradation and dilution in cell division) | 0.004 s^-1^ | Section 1.7.1 |
| **k_d_^P^** | Protein decay rate (degradation and dilution in cell division) | 8.65×10^-5^ s^-1^ | Section 1.7.1 |
| **k_70_^holo^** | Rate of formation of a RNAP.σ^70^ holoenzyme | 0.001 s^-1^ | Section 1.7.3 |
| **k_70_^core^** | Rate of unbinding of a RNAP and a σ^70^ | 0.001 s^-1^ | Section 1.7.3 |
| **k_38_^holo^** | Rate of formation of a RNAP.σ^38^ holoenzyme | 0.0011 s^-1^ | Section 1.7.3 |
| **k_38_^core^** | Rate of unbinding of a RNAP and a σ^38^ | 0.001 s^-1^ | Section 1.7.3 |
| **RNAP** | Initial amount of RNAP core enzymes | 144 | Section 1.7.2 |
| **σ^70^** | Initial amount of σ^70^ | 288 | Section 1.7.3 |
| **σ^38^** | Initial amount of σ^38^ | 0 | Section 1.7.3 |

**Table C:** Parameter values for the bimodal model in the stationary growth phase.

| **Parameter** | **Value** | **Justification** |
| --- | --- | --- |
| **k_L_** | 0.00001 s^-1^ | No changes assumed when cells transition from exponential to stationary growth phase. |
| **k_H_** | 0.00001 s^-1^ |  |
| **k_bind_^L^** | 1.54×10^-5^ s^-1^ |  |
| **k_esc_^L^** | 2.21×10^-3^ s^-1^ |  |
| **k_bind_^H^** | 1.15×10^-4^ s^-1^ |  |
| **k_esc_^H^** | 1.66×10^-2^ s^-1^ |  |
| **k_tr_** | 1.1×10^-3^ s^-1^ | Translation activity has been reported to decrease 40 times when cells enter stationary phase [23] |
| **k_d_^RNA^** | 2.14×10^-3^ s^-1^ | Average lifetime of mRNA of 7.8 min [12] and a dilution rate of 4.24×10^-4^ s^-1^ (Fig U in S1 Text). |
| **k_d_^P^** | 3.02×10^-5^ s^-1^ | Protein degradation of 8%/h [13] and a dilution rate of 4.24×10^-4^ s^-1^ (Fig U in S1 Text). |
| **k_70_^holo^** | 0.001 s^-1^ | No changes assumed when cells transition from exponential to stationary growth phase. |
| **k_70_^core^** | 0.001 s^-1^ |  |
| **k_38_^holo^** | 0.0011 s^-1^ |  |
| **k_38_^core^** | 0.001 s^-1^ |  |
| **RNAP** | 144 | RNAP and σ^70^ numbers remain relatively constant between exponential and stationary phase. |
| **σ^70^** | 288 |  |
| **σ^38^** | 63 | During the stationary growth phase, the ratio [σ^38^]/[σ^70^] is ≈22%. (Section 1.7.3) |

**Table D:** Parameter values for a reference positive feedback loop model. We ran simulations for rates constants 10, 5, and 2× lower as well as for 2, 5, and 10× higher. The values were selected to be within realistic ranges of parameters values, while also allowing for easy visualization of the influence of each parameter on the distribution.

| **Parameter** | **Parameter Description** | **Value** |
| --- | --- | --- |
| **k_70_^holo^** | Rate of formation of a RNAP.σ^70^ holoenzyme | 0.001 s^-1^ |
| **k_70_^core^** | Rate of unbinding of a RNAP and a σ^70^ | 0.001 s^-1^ |
| **k_bind_^spurious^** | Rate of spurious promoter binding rate | 3×10^-5^ s^-1^ |
| **k_esc_^spurious^** | Rate of spurious promoter escape | 3×10^-5^ s^-1^ |
| **k_act_** | Rate of binding of the protein to the promoter region (activating the gene) | 0.15 s^-1^ |
| **k_inact_** | Rate of unbinding of the protein to the promoter region (inactivating the gene) | 1 s^-1^ |
| **k_bind_** | Rate of promoter binding rate when gene is active | 1.15×10^-4^ s^-1^ |
| **k_esc_** | Rate of promoter escape rate when gene is active | 1.66×10^-2^ s^-1^ |
| **k_tr_** | Translation rate | 0.047 s^-1^ |
| **k_d_^RNA^** | RNA decay rate (degradation and dilution in cell division) | 0.004 s^-1^ |
| **k_d_^P^** | Protein decay rate (degradation and dilution in cell division) | 8.65×10^-5^ s^-1^ |
| **RNAP** | Initial amount of RNAP core enzymes | 144 |
| **σ^70^** | Initial amount of σ^70^ | 288 |

**Table E:** Strains used in the study.

| **Strain Name** | **Genotype** | **Source** | **Gene Tagged** |
| --- | --- | --- | --- |
| SX1835 | F-, araB791-YFP(::cat), Δ(argF-lac)169, gal-490, Δ(modF-ybhJ)803, λ[cI857 Δ(cro-bioA)], IN(rrnD-rrnE)1, rph-1 | Yale CGSC (CGSC # 13390) | araB |
| SX1843 | F-, Δ(argF-lac)169, gal-490, Δ(modF-ybhJ)803, λ[cI857 Δ(cro-bioA)], IN(rrnD-rrnE)1, bcsE792-YFP(::cat), rph-1 | Yale CGSC (CGSC # 13398) | bcse |
| SX1747 | F-, Δ(argF-lac)169, cusS791-YFP(::cat), gal-490, Δ(modF-ybhJ)803, λ[cI857 Δ(cro-bioA)], IN(rrnD-rrnE)1, rph-1 | Yale CGSC (CGSC # 13302) | cuss |
| SX1798 | F-, Δ(argF-lac)169, gal-490, Δ(modF-ybhJ)803, λ[cI857 Δ(cro-bioA)], cydD793-YFP(::cat), IN(rrnD-rrnE)1, rph-1 | Yale CGSC (CGSC # 13353) | cydd |
| SX1800 | F-, Δ(argF-lac)169, cyoD792-YFP(::cat), gal-490, Δ(modF-ybhJ)803, λ[cI857 Δ(cro-bioA)], IN(rrnD-rrnE)1, rph-1 | Yale CGSC (CGSC # 13355) | cyod |
| SX1045 | F-, Δ(argF-lac)169, gal-490, Δ(modF-ybhJ)803, λ[cI857 Δ(cro-bioA)], IN(rrnD-rrnE)1, dut-791-YFP(::cat), rph-1 | Yale CGSC (CGSC # 12600) | dut |
| SX1912 | F-, Δ(argF-lac)169, gal-490, Δ(modF-ybhJ)803, λ[cI857 Δ(cro-bioA)], edd-791-YFP(::cat), IN(rrnD-rrnE)1, rph-1 | Yale CGSC (CGSC # 13461) | edd |
| SX1742 | F-, fhuA791-YFP(::cat), Δ(argF-lac)169, gal-490, Δ(modF-ybhJ)803, λ[cI857 Δ(cro-bioA)], IN(rrnD-rrnE)1, rph-1 | Yale CGSC (CGSC # 13297) | fhuA |
| SX1625 | F-, frsA791-YFP(::cat), Δ(argF-lac)169, gal-490, Δ(modF-ybhJ)803, λ[cI857 Δ(cro-bioA)], IN(rrnD-rrnE)1, rph-1 | Yale CGSC (CGSC # 13180) | frsA |
| SX1778 | F-, Δ(argF-lac)169, gal-490, Δ(modF-ybhJ)803, λ[cI857 Δ(cro-bioA)], fucK791-YFP(::cat), IN(rrnD-rrnE)1, rph-1 | Yale CGSC (CGSC # 13333) | fucK |
| SX1100 | F-, Δ(argF-lac)169, gal-490, Δ(modF-ybhJ)803, λ[cI857 Δ(cro-bioA)], IN(rrnD-rrnE)1, lldD791-YFP(::cat), rph-1 | Yale CGSC (CGSC # 12655) | lldD |
| SX1447 | F-, Δ(argF-lac)169, gal-490, Δ(modF-ybhJ)803, λ[cI857 Δ(cro-bioA)], IN(rrnD-rrnE)1, malP792-YFP(::cat), rph-1 | Yale CGSC (CGSC # 13002) | malP |
| SX1454 | F-, Δ(argF-lac)169, gal-490, Δ(modF-ybhJ)803, λ[cI857 Δ(cro-bioA)], manX792-YFP(::cat), IN(rrnD-rrnE)1, rph-1 | Yale CGSC (CGSC # 13009) | manX |
| SX1507 | F-, Δ(argF-lac)169, gal-490, Δ(modF-ybhJ)803, λ[cI857 Δ(cro-bioA)], narG791-YFP(::cat), IN(rrnD-rrnE)1, rph-1 | Yale CGSC (CGSC # 13602) | narG |
| SX1347 | F-, Δ(argF-lac)169, gal-490, Δ(modF-ybhJ)803, λ[cI857 Δ(cro-bioA)], paaX796-YFP(::cat), IN(rrnD-rrnE)1, rph-1 | Yale CGSC (CGSC # 12902) | paaX |
| SX1618 | F-, rluA791-YFP(::cat), Δ(argF-lac)169, gal-490, Δ(modF-ybhJ)803, λ[cI857 Δ(cro-bioA)], IN(rrnD-rrnE)1, rph-1 | Yale CGSC (CGSC # 13173) | rluA |
| SX1971 | F-, Δ(argF-lac)169, gal-490, Δ(modF-ybhJ)803, λ[cI857 Δ(cro-bioA)], IN(rrnD-rrnE)1, zntA791-YFP(::cat), rph-1 | Yale CGSC (CGSC # 13526) | zntA |
| SX1105 | F-, Δ(argF-lac)169, gal-490, Δ(modF-ybhJ)803, λ[cI857 Δ(cro-bioA)], zwf-791-YFP(::cat), IN(rrnD-rrnE)1, rph-1 | Yale CGSC (CGSC # 12660) | zwf |
| SX1766 | F-, Δ(argF-lac)169, gal-490, Δ(modF-ybhJ)803, λ[cI857 Δ(cro-bioA)], baeR791-YFP(::cat), IN(rrnD-rrnE)1, rph-1 | Yale CGSC (CGSC # 13321) | baeR |
| SX1475 | F-, Δ(argF-lac)169, gal-490, Δ(modF-ybhJ)803, λ[cI857 Δ(cro-bioA)], dhaK791-YFP(::cat), IN(rrnD-rrnE)1, rph-1 | Yale CGSC (CGSC # 13030) | dhaK |
| SX1695 | F-, Δ(argF-lac)169, gal-490, Δ(modF-ybhJ)803, λ[cI857 Δ(cro-bioA)], elaB792-YFP(::cat), IN(rrnD-rrnE)1, rph-1 | Yale CGSC (CGSC # 13250) | elaB |
| SX2019 | F-, Δ(argF-lac)169, gal-490, Δ(modF-ybhJ)803, λ[cI857 Δ(cro-bioA)], fbaB792-YFP(::cat), IN(rrnD-rrnE)1, rph-1 | Yale CGSC (CGSC # 13574) | fbaB |
| SX1535 | F-, ldcC791-YFP(::cat), Δ(argF-lac)169, gal-490, Δ(modF-ybhJ)803, λ[cI857 Δ(cro-bioA)], IN(rrnD-rrnE)1, rph-1 | Yale CGSC (CGSC # 13090) | ldcC |
| SX1526 | F-, Δ(argF-lac)169, gal-490, Δ(modF-ybhJ)803, λ[cI857 Δ(cro-bioA)], katE791-YFP(::cat), IN(rrnD-rrnE)1, rph-1 | Yale CGSC (CGSC # 13081) | katE |
| SX1600 | F-, Δ(argF-lac)169, gal-490, Δ(modF-ybhJ)803, λ[cI857 Δ(cro-bioA)], mdtA791-YFP(::cat), IN(rrnD-rrnE)1, rph-1 | Yale CGSC (CGSC # 13155) | mdtA |
| SX1437 | F-, Δ(argF-lac)169, gal-490, Δ(modF-ybhJ)803, λ[cI857 Δ(cro-bioA)], ppk-791-YFP(::cat), IN(rrnD-rrnE)1, rph-1 | Yale CGSC (CGSC # 12992) | ppk |
| SX1441 | F-, Δ(argF-lac)169, gal-490, Δ(modF-ybhJ)803, λ[cI857 Δ(cro-bioA)], IN(rrnD-rrnE)1, rph-1, ravA791-YFP(::cat) | Yale CGSC (CGSC # 12996) | ravA |
| SX1641 | F-, Δ(argF-lac)169, gal-490, Δ(modF-ybhJ)803, λ[cI857 Δ(cro-bioA)], rssA791-YFP(::cat), IN(rrnD-rrnE)1, rph-1 | Yale CGSC (CGSC # 13196) | rssA |
| SX1952 | F-, Δ(argF-lac)169, gal-490, Δ(modF-ybhJ)803, λ[cI857 Δ(cro-bioA)], speC791-YFP(::cat), IN(rrnD-rrnE)1, rph-1 | Yale CGSC (CGSC # 13507) | speC |
| SX1956 | F-, Δ(argF-lac)169, gal-490, Δ(modF-ybhJ)803, λ[cI857 Δ(cro-bioA)], treA791-YFP(::cat), IN(rrnD-rrnE)1, rph-1 | Yale CGSC (CGSC # 13511) | treA |
| SX1524 | F-, Δ(argF-lac)169, gal-490, Δ(modF-ybhJ)803, λ[cI857 Δ(cro-bioA)], IN(rrnD-rrnE)1, rph-1, viaA791-YFP(::cat) | Yale CGSC (CGSC # 13079) | viaA |
| SX1718 | F-, Δ(argF-lac)169, gal-490, Δ(modF-ybhJ)803, λ[cI857 Δ(cro-bioA)], wrbA791-YFP(::cat), IN(rrnD-rrnE)1, rph-1 | Yale CGSC (CGSC # 13273) | wrbA |
| SX1253 | F-, Δ(argF-lac)169, gal-490, Δ(modF-ybhJ)803, λ[cI857 Δ(cro-bioA)], xthA791-YFP(::cat), IN(rrnD-rrnE)1, rph-1 | Yale CGSC (CGSC # 12808) | xthA |
| SX1973 | F-, Δ(argF-lac)169, gal-490, Δ(modF-ybhJ)803, λ[cI857 Δ(cro-bioA)], ybjP792-YFP(::cat), IN(rrnD-rrnE)1, rph-1 | Yale CGSC (CGSC # 13528) | ybjP |
| SX1975 | F-, Δ(argF-lac)169, gal-490, Δ(modF-ybhJ)803, λ[cI857 Δ(cro-bioA)], yccJ791-YFP(::cat), IN(rrnD-rrnE)1, rph-1 | Yale CGSC (CGSC # 13530) | yccJ |
| SX1450 | F-, Δ(argF-lac)169, gal-490, Δ(modF-ybhJ)803, λ[cI857 Δ(cro-bioA)], ydhV795-YFP(::cat), IN(rrnD-rrnE)1, rph-1 | Yale CGSC (CGSC # 13005) | ydhV |
| SX1439 | F-, Δ(argF-lac)169, gal-490, Δ(modF-ybhJ)803, λ[cI857 Δ(cro-bioA)], ydhW796-YFP(::cat), IN(rrnD-rrnE)1, rph-1 | Yale CGSC (CGSC # 12994) | ydhW |
| SX1733 | F-, Δ(argF-lac)169, gal-490, Δ(modF-ybhJ)803, λ[cI857 Δ(cro-bioA)], IN(rrnD-rrnE)1, yhfG791-YFP(::cat), rph-1 | Yale CGSC (CGSC # 13288) | yhfG |
| SX1435 | F-, Δ(argF-lac)169, gal-490, Δ(modF-ybhJ)803, λ[cI857 Δ(cro-bioA)], IN(rrnD-rrnE)1, rph-1, aidB791-YFP(::cat) | Yale CGSC (CGSC # 12990) | aidB |
| SX1267 | F-, Δ(argF-lac)169, gal-490, Δ(modF-ybhJ)803, λ[cI857 Δ(cro-bioA)], alkA791-YFP(::cat), IN(rrnD-rrnE)1, rph-1 | Yale CGSC (CGSC # 12822) | alkA |
| SX1793 | F-, Δ(argF-lac)169, gal-490, Δ(modF-ybhJ)803, λ[cI857 Δ(cro-bioA)], appC791-YFP(::cat), IN(rrnD-rrnE)1, rph-1 | Yale CGSC (CGSC # 13348) | appC |
| SX1791 | F-, Δ(argF-lac)169, gal-490, Δ(modF-ybhJ)803, λ[cI857 Δ(cro-bioA)], IN(rrnD-rrnE)1, rph-1, cpxR791-YFP(::cat) | Yale CGSC (CGSC # 13346) | cpxR |
| SX1551 | F-, Δ(argF-lac)169, gal-490, Δ(modF-ybhJ)803, λ[cI857 Δ(cro-bioA)], gltB792-YFP(::cat), IN(rrnD-rrnE)1, rph-1 | Yale CGSC (CGSC # 13348) | gltB |
| SX1541 | F-, Δ(argF-lac)169, gal-490, Δ(modF-ybhJ)803, λ[cI857 Δ(cro-bioA)], gltD793-YFP(::cat), IN(rrnD-rrnE)1, rph-1 | Yale CGSC (CGSC # 13096) | gltD |
| SX1675 | F-, Δ(argF-lac)169, gal-490, Δ(modF-ybhJ)803, λ[cI857 Δ(cro-bioA)], IN(rrnD-rrnE)1, gor-791-YFP(::cat), rph-1 | Yale CGSC (CGSC # 13230) | gor |
| SX1458 | F-, Δ(argF-lac)169, gal-490, Δ(modF-ybhJ)803, λ[cI857 Δ(cro-bioA)], metK794-YFP(::cat), IN(rrnD-rrnE)1, rph-1 | Yale CGSC (CGSC # 13031) | metK |
| SX1767 | F-, Δ(argF-lac)169, gal-490, Δ(modF-ybhJ)803, λ[cI857 Δ(cro-bioA)], mlrA791-YFP(::cat), IN(rrnD-rrnE)1, rph-1 | Yale CGSC (CGSC # 13322) | mlrA |
| SX1568 | F-, Δ(argF-lac)169, gal-490, Δ(modF-ybhJ)803, λ[cI857 Δ(cro-bioA)], IN(rrnD-rrnE)1, rph-1, pgi-791-YFP(::cat) | Yale CGSC (CGSC # 13123) | pgi |
| SX1571 | F-, Δ(argF-lac)169, gal-490, Δ(modF-ybhJ)803, λ[cI857 Δ(cro-bioA)], IN(rrnD-rrnE)1, rph-1, pstS793-YFP(::cat) | Yale CGSC (CGSC # 13126) | pstS |
| SX1960 | F-, Δ(argF-lac)169, gal-490, Δ(modF-ybhJ)803, λ[cI857 Δ(cro-bioA)], IN(rrnD-rrnE)1, rph-1, oxyR791-YFP(::cat) | Yale CGSC (CGSC # 13515) | oxyR |
| SX1902 | F-, Δ(argF-lac)169, gal-490, Δ(modF-ybhJ)803, λ[cI857 Δ(cro-bioA)], IN(rrnD-rrnE)1, aldB792-YFP(::cat), rph-1 | Yale CGSC (CGSC # 13457) | aldB |
| SX1506 | F-, Δ(argF-lac)169, gal-490, Δ(modF-ybhJ)803, λ[cI857 Δ(cro-bioA)], dps-791-YFP(::cat), IN(rrnD-rrnE)1, rph-1 | Yale CGSC (CGSC # 13061) | dps |
| SX1662 | F-, Δ(argF-lac)169, gal-490, Δ(modF-ybhJ)803, λ[cI857 Δ(cro-bioA)], IN(rrnD-rrnE)1, rph-1, osmY792-YFP(::cat) | Yale CGSC (CGSC # 13217) | osmY |
| SX1067 | F-, pyrH791-YFP(::cat), Δ(argF-lac)169, gal-490, Δ(modF-ybhJ)803, λ[cI857 Δ(cro-bioA)], IN(rrnD-rrnE)1, rph-1 | Yale CGSC (CGSC # 12622) | pyrH |
| SX1358 | F-, Δ(argF-lac)169, gal-490, Δ(modF-ybhJ)803, λ[cI857 Δ(cro-bioA)], rcsD793-YFP(::cat), IN(rrnD-rrnE)1, rph-1 | Yale CGSC (CGSC # 12913) | rcsD |
| SX1123 | F-, Δ(argF-lac)169, gal-490, Δ(modF-ybhJ)803, λ[cI857 Δ(cro-bioA)], torA791-YFP(::cat), IN(rrnD-rrnE)1, rph-1 | Yale CGSC (CGSC # 12678) | torA |

**Supplementary References**

1. Taniguchi Y, Choi PJ, Li G-W, Chen H, Babu M, Hearn J, et al. Quantifying E. coli Proteome and Transcriptome with Single-Molecule Sensitivity in Single Cells. Science. 2010;329: 533–538. doi:10.1126/science.1188308

2. Zaslaver A, Bren A, Ronen M, Itzkovitz S, Kikoin I, Shavit S, et al. A comprehensive library of fluorescent transcriptional reporters for Escherichia coli. Nat Methods. 2006;3: 623–628. doi:10.1038/nmeth895

3. Tukey JW. Schematic summaries: Fences, and outside values. Exploratory data analysis. Reading, Massachusetts: Addison-Wesley Publishing; 1977. pp. 43–44.

4. Chong S, Chen C, Ge H, Xie XS. Mechanism of transcriptional bursting in bacteria. Cell. 2014;158: 314–326. doi:10.1016/j.cell.2014.05.038

5. Almeida BLB, M Bahrudeen MN, Chauhan V, Dash S, Kandavalli V, Häkkinen A, et al. The transcription factor network of E. coli steers global responses to shifts in RNAP concentration. Nucleic Acids Res. 2022. doi:10.1093/nar/gkac540

6. Häkkinen A, Muthukrishnan A-B, Mora A, Fonseca JM, Ribeiro AS. CellAging: a tool to study segregation and partitioning in division in cell lineages of Escherichia coli. Bioinformatics. 2013;29: 1708–1709. doi:10.1093/bioinformatics/btt194

7. Mora AD, Vieira PM, Manivannan A, Fonseca JM. Automated drusen detection in retinal images using analytical modelling algorithms. Biomed Eng Online. 2011;10: 59. doi:10.1186/1475-925X-10-59

8. Schilling MF, Watkins AE, Watkins W. Is Human Height Bimodal? Am Stat. 2002;56: 223–229. doi:10.1198/00031300265

9. Raftery AE. Bayesian Model Selection in Social Research. Sociol Methodol. 1995;25: 111–163. doi:10.2307/271063

10. Lloyd-Price J, Gupta A, Ribeiro AS. SGNS2: A compartmentalized stochastic chemical kinetics simulator for dynamic cell populations. Bioinformatics. 2012;28: 3004–3005. doi:10.1093/bioinformatics/bts556

11. Gillespie DT. Exact stochastic simulation of coupled chemical reactions. J Phys Chem. 1977;81: 2340–2361. doi:10.1021/j100540a008

12. Chen, Shiroguchi K, Ge H, Xie XS. Genome-wide study of mRNA degradation and transcript elongation in Escherichia coli. Mol Syst Biol. 2015;11: 781. doi:10.15252/msb.20145794

13. Maurizi MR. Proteases and protein degradation in Escherichia coli. Experientia. 1992;48: 178–201. doi:10.1007/BF01923511

14. Bremer H, Dennis P, Ehrenberg M. Free RNA polymerase and modeling global transcription in Escherichia coli. Biochimie. 2003;85: 597–609. doi:10.1016/S0300-9084(03)00105-6

15. Maeda H, Fujita N, Ishihama A. Competition among seven Escherichia coli σ subunits: Relative binding affinities to the core RNA polymerase. Nucleic Acids Res. 2000;28: 3497–3503. doi:10.1093/nar/28.18.3497

16. Shepherd N, Dennis P, Bremer H. Cytoplasmic RNA polymerase in Escherichia coli. J Bacteriol. 2001;183: 2527–2534. doi:10.1128/JB.183.8.2527-2534.2001

17. Kandavalli VK, Tran H, Ribeiro AS. Effects of σ factor competition are promoter initiation kinetics dependent. Biochim Biophys Acta. 2016;1859: 1281–1288. doi:10.1016/j.bbagrm.2016.07.011

18. Ishihama A. Functional modulation of Escherichia coli RNA polymerase. Annu Rev Microbiol. 2000;54: 499–518. doi:10.1146/annurev.micro.54.1.499

19. Piper SE, Mitchell JE, Lee DJ, Busby SJW. A global view of Escherichia coli Rsd protein and its interactions. Mol Biosyst. 2009;5: 1943–1947. doi:10.1039/B904955J

20. Mauri M, Klumpp S. A Model for Sigma Factor Competition in Bacterial Cells. PLoS Comput Biol. 2014;10: e1003845. doi:10.1371/journal.pcbi.1003845

21. Lu P, Vogel C, Wang R, Yao X, Marcotte EM. Absolute protein expression profiling estimates the relative contributions of transcriptional and translational regulation. Nat Biotechnol. 2007;25: 117–124. doi:10.1038/nbt1270

22. Kaplan D. Knee Point. In: MATLAB Central File Exchange [Internet]. 16 Feb 2012 [cited 26 Oct 2022]. Available: https://se.mathworks.com/matlabcentral/fileexchange/35094-knee-point

23. Reier K, Liiv A, Remme J. Ribosome Protein Composition Mediates Translation during the Escherichia coli Stationary Phase. Int J Mol Sci. 2023;24: 3128. doi:10.3390/ijms24043128
